# A critical assessment of marine predator isoscapes within the southern Indian Ocean

**DOI:** 10.1186/s40462-020-00208-8

**Published:** 2020-06-29

**Authors:** Tegan Carpenter-Kling, Pierre Pistorius, Ryan Reisinger, Yves Cherel, Maëlle Connan

**Affiliations:** 1 Marine Apex Predator Research Unit (MAPRU), Department of Zoology, Institute for Coastal and Marine Research, Nelson Mandela University, Port Elizabeth, South Africa; 2 DST-NRF Centre of Excellence at the FitzPatrick Institute of African Ornithology, Nelson Mandela University, Port Elizabeth, South Africa; 3 Centre d’Etudes Biologiques de Chizé, UMR 7372 du CNRS-La Rochelle Université, 79360 Villiers-en-Bois, France

**Keywords:** Geolocation, Stable isotope ecology, Southern Ocean, Seabirds, Procellariiformes, Penguins

## Abstract

**Background:**

Precise and accurate retrospective geolocation of marine predators via their tissues’ isotopic composition relies on quality reference maps of relevant isotopic gradients (“isoscapes”). Additionally, a good working knowledge of any discrimination factors that may offset a marine predator’s isotopic composition from baseline isotopic values, as well as tissue specific retention rates, are imperative. We provide a critical assessment of inter-specific differences among marine predator-level isoscapes within the Indian Sector of the Southern Ocean.

**Methods:**

We combined fine-scale GPS tracking data and concurrent blood plasma δ^13^C and δ^15^N values of eight seabird species (three albatross, two giant petrel and three penguin species) breeding at Marion Island to produce species- and guild-specific isoscapes.

**Results:**

Overall, our study revealed latitudinal spatial gradients in both δ^13^C and δ^15^N for far-ranging seabirds (albatrosses and giant petrels) as well as inshore-offshore gradients for near-ranging seabirds (penguins). However, at the species level, latitudinal spatial gradients were not reflected in the δ^13^C and δ^15^N isoscapes of two and three, respectively, of the five far-ranging species studied. It is therefore important when possible to estimate and apply species-specific isoscapes or have a good understanding of any factors and pathways affecting marine predators’ isotopic composition when estimating the foraging distribution of marine predators via their tissues’ stable isotope compositions.

**Conclusions:**

Using a multi-species approach, we provide evidence of large and regional scale systematic spatial variability of δ^13^C and δ^15^N at the base of the marine food web that propagates through trophic levels and is reflected in the isotopic composition of top predators’ tissues.

## Background

Some of the greatest threats faced by land-breeding marine predators are experienced at sea. These include bycatch-risk and changes in food availability as a result of competition with fisheries and climate change [1–3]. Therefore, to implement effective conservation-based marine spatial planning there is a growing need to better understand the at-sea distribution of marine predators [4–6]. This has led to an impressive growth in the number of tracking studies in recent years (reviewed in [7]), often with the general aim of providing policy-relevant information on important habitat for the respective study species [8]. However, dataloggers are still cumbersome for small species (e.g. some burrowing seabird species) and deployment of loggers on study animals requires significant amounts of time in the field, especially when instruments need to be retrieved. Stable isotope ecology as a tool for retrospective geolocation of predator foraging grounds has relatively recently emerged as an alternative and complimentary method to conventional tracking studies [9, 10]. Stable isotope analysis of body tissues is relatively cheap, less demanding in terms of field time and as a result allows for easy sampling of a greater number of individuals than would often be incorporated in tracking studies (e.g. [11]).

The precision and accuracy of retrospective geolocation of marine predators based on their isotopic composition is primarily reliant on two important factors. Firstly, the reliance on the availability of reference maps of the relevant isotopic gradients, known as “isoscapes” [12]. In marine systems, marine predator movement is commonly inferred by linking the ratios of the stable isotopes of carbon (^13^C/^12^C; δ^13^C), and to a lesser extent nitrogen (^15^N/^14^N; δ^15^N), of their tissues to known gradients of δ^13^C and δ^15^N values present at the base of their food webs (e.g. [13–15]). Across the global oceans, there is a strong negative latitudinal gradient in the δ^13^C values of phytoplankton, from the equator towards the poles [16], as well as from inshore benthic habitats to offshore pelagic habitats [17–19]. Whereas gradients of δ^15^N are not as strong or predictable, the δ^15^N values of phytoplankton tend to be lower or higher in areas of nitrogen fixation (e.g. pelagic oceans) or denitrification (e.g. upwelling regions around coastlines), respectively [20, 21]. Secondly, a good working knowledge of potential discrimination factors which may offset a consumer’s isotopic composition from baseline isotopic values is required. These discrimination factors may vary with diet composition, isotopic averaging as well as physiological fractionation through intermediate trophic levels, isotopic turnover rates and physiological transformation in the consumer [9, 12]. This includes tissue-specific retention times, as isotopic turnover of different tissues varies greatly [22].

Previously, studies which estimated oceanic δ^13^C and δ^15^N isoscapes have largely used organisms close to the base of the food web (e.g. [16, 23, 24]) or particulate organic matter [25–27]. The isotope ratios of organisms near the base of the food web (e.g. phytoplankton) or particulate organic matter are influenced by broad scale and localized chemical element circulation and physical oceanographic features [28]. Furthermore, due to the high turnover rate of these organisms, their isotope ratios may change daily [29]. However, due to temporal integration of isotopic ratios from the base of the web through to higher predators [30], the stable isotopic compositions of marine predator tissues are not expected to reflect these short-term changes in the baseline but rather reflect more consistent isotope gradients.

The estimation of δ^13^C and δ^15^N predator-specific isoscapes requires information on the movement of the predator during the time which the body tissue of interest incorporated its isotope composition (e.g. [14, 31]). While breeding, marine predators such as seabirds are central place foragers, regularly returning to their nest to provide care to their offspring [32]. Therefore, due to the ease of recapture of individuals after single foraging trips and collection of tissues for stable isotope analysis, seabirds represent ideal study species to investigate inter-specific differences in marine predator-level δ^13^C and δ^15^N isoscapes. In addition, the dichotomy in foraging mode presented in seabirds, and their associated foraging constraints, provides an opportunity to compare flying (e.g. albatrosses and giant petrels), and diving (penguins) seabirds. The vast inter-specific differences in the at-sea foraging distribution of seabirds furthermore allow for both the investigation of isoscapes over geographically extensive areas as well as inter-specific differences in isoscapes within locations utilised by multiple species.

Several studies have attempted to reconstruct δ^13^C and δ^15^N predator-specific isoscapes by combining known location and distributional range [17] or movement of seabirds [11, 14, 31, 33, 34] to temporally matched tissue isotopic values. The blood plasma of wild birds has a half-life of a few days and it is generally assumed that δ^13^C and δ^15^N values of seabird blood plasma reflects approximately 7 days bioaccumulation of isotope values of prey prior to sampling [22, 35]. This makes it an ideal tissue to link to fine-scale tracking data as the movements of the birds can be known with high accuracy several days prior to sampling. However, integrating concurrently collected δ^13^C and δ^15^N blood plasma values with fine-scale tracking data has rarely been done and only in a single-species context [14, 31]. Here, we combine fine-scale GPS tracking data and concurrent blood plasma isotopic values of five Procellariiformes (albatrosses and giant petrels) and three Sphenisciformes (penguins) species to produce species and guild specific δ^13^C and δ^15^N isoscapes within the Southern Indian Ocean. We assigned an individual’s isotopic values to a time integrated mean foraging location and use these data to investigate spatial patterns of a species δ^13^C and δ^15^N plasma values. The study aims to investigate the influence of species on the determination of δ^13^C and δ^15^N isoscapes as well as differences between predators with different movement modes (flying vs. diving). We hypothesized that the δ^13^C and δ^15^N tissue values of most species exhibit a spatial gradient and the extent and strength of this gradient is dependent on the species foraging range with the following predictions:

1. *Influence of seabird species on the detection of large-scale spatial variability in δ*^*13*^*C and δ*^*15*^*N isoscapes.* We predict that due to the vast distances travelled by flying species during a single foraging trip, their δ^13^C and δ^15^N tissue values will reflect known baseline latitudinal δ^13^C and δ^15^N gradients (e.g. [16, 20]). However, due to sharp changes in baseline δ^13^C values at frontal zones [25], we predict that if individuals of a species do not cross different major fronts within the Indian sector of the Southern Ocean, the δ^13^C values of that species’ tissues will not reflect a spatial gradient.
2. *δ*^*13*^*C and δ*^*15*^*N isotopic values of major fronts and water zones within seabird species isoscapes.* We predict that within the isoscapes of species which crossed one or more major fronts, there will be consistent stepwise increases at major fronts from south to north.
3. *Influence of seabird species on the detection of fine scale spatial variability in δ*^*13*^*C and δ*^*15*^*N isoscapes.* Previously, it has been shown that an inshore/offshore and benthic/pelagic effect can be detected in the isotopic values of seabirds [17, 36, 37]. Thus, due to the more limited foraging range of penguins compared to that of the flying species in this study, we predict that the δ^13^C and δ^15^N plasma values of penguins will not reflect a latitudinal gradient, but will rather reflect the inshore/offshore and benthic/pelagic foraging habitat of the species.

## Materials and methods

### Study site and species

The Prince Edward Archipelago is located in the Indian sector of the Southern Ocean between the sub-Antarctic and Antarctic polar fronts [38]. Ascending from a depth of approximately 3000 m, the archipelago consists of two islands, Marion Island (~ 240 km^2^) and Prince Edward Island (~ 45 km^2^), which are located 19 km apart and separated by a shallow inter-island shelf that ranges from 40 to 400 m in depth [39]. These two islands provide breeding grounds for more than five million seabirds and seals [40].

This study includes three of the four albatross species (wandering *Diomedea exulans*, grey-headed *Thalassarche chrysostoma* and sooty *Phoebetria fusca* albatrosses), both giant petrel species (northern *Macronectes halli* and southern *M. giganteus* giant petrels) and three of the four penguin species (gentoo *Pygoscelis papua*, macaroni *Eudyptes chrysolophus* and eastern rockhopper *E. filholi* penguins) breeding at Marion Island.

### Data collection

Field work was conducted between August to March 2015/16–2017/18, along the south-east coast of Marion Island (46°54′S; 37°45′E) during the breeding seasons of the respective study species (Supplementary Material S1). GPS data loggers (CatLog-S GPS loggers, Perthold Engineering LLC USA, 50 × 22 × 8 mm, ~ 24.5 g) were deployed on individuals that were either incubating or brooding small chicks. In total, loggers were deployed on 176 individuals, which included 32 wandering, 28 grey-headed and 22 sooty albatrosses, 27 northern and 23 southern giant petrels and 17 gentoo, 18 macaroni and 9 eastern rockhopper penguins. Loggers were set to record geographic locations at hourly intervals for flying birds (i.e. albatrosses and giant petrels) and two-minute intervals for penguins.

Loggers were retrieved after 1.2 ± 0.5 foraging trips for flying birds and 1.7 ± 0.9 foraging trips for penguins (Table 1). Upon retrieval of GPS loggers, ~ 1 ml of blood was collected from the tarsal vein of flying birds or the brachial vein of penguins using a sterile heparinised 25 gauge needle. Approximately 0.5 ml of the blood was centrifuged within 3 – 4 h after collection, separated into red blood cells and plasma, stored in 70% ethanol and frozen until preparation for stable isotope analysis [41].

**Table 1:** Delipidated or normalized plasma δ^13^C and raw plasma δ^15^N values of albatrosses, giant petrels, and penguins breeding at Marion Island, which were tracked simultaneously with GPS data loggers during 2015–2018. Number of individuals indicates number of birds with tracks and corresponding stable isotopic values. Number of tracks indicates number of tracks used to estimate mean foraging locations as multiple tracks were recorded for brooding individuals (See Materials and Methods). Values given as mean ± SD (range)

Common name	Number of individuals	Number of tracks per species	Number of tracks per individual	δ^13^C (‰)	δ^15^N (‰)
**Albatrosses**					
Wandering	32	32	1.0 ± 0.0 (1; 1)	−20.7 ± 1.5 (−23.6; −18.4)	14.0 ± 1.0 (12.1; 15.5)
Grey-headed	28	36	1.3 ± 0.5 (1; 2)	− 20.2 ± 1.0 (−22.0; − 18.5)	11.4 ± 0.4 (10.4; 12.4)
Sooty	22	36	1.6 ± 0.7 (1; 3)	−20.8 ± 0.7 (− 22.2; − 19.8)	12.0 ± 0.4 (11.4; 13.1)
**Giant petrels**					
Northern	27	29	1.1 ± 0.3 (1; 2)	−19.8 ± 1.2 (− 22.7; − 17.1)	14.4 ± 0.8 (12.7; 15.9)
Southern	23	25	1.1 ± 0.3 (1; 2)	−22.7 ± 0.5 (− 23.6; − 21.8)	12.8 ± 0.4 (12.3; 13.4)
**Penguins**					
Gentoo	17	26	1.5 ± 0.9 (1; 4)	− 21.7 ± 0.6 (− 23.2; − 20.9)	10.2 ± 0.5 (9.5; 11.4)
Macaroni	18	29	1.6 ± 0.8 (1; 3)	−22.3 ± 0.2 (− 22.7; − 22.0)	8.8 ± 0.3 (8.0; 9.2)
Rockhopper	9	19	2.1 ± 1.1 (1; 4)	−22.6 ± 0.2 (− 22.9; − 22.1)	8.6 ± 0.4 (7.7; 9.1)

### GPS analysis

A foraging trip was defined as the last location on land until the first location back on land. Land-based locations were removed for further analysis. Unrealistic GPS locations were identified and removed using a speed filter algorithm (R package *trip*; [42]). Unrealistic locations were identified as those requiring movement speeds greater than 135 km h^− 1^ [32] for flying birds and greater than 10 km h^− 1^ for penguins [43]. Post-filtering, positions were linearly interpolated at one-hour intervals for the flying birds’ trips (R package *adehabitatLT;* [44]). The filtered data for penguins were processed using a continuous-time correlated random walk (CRAWL) model to estimate the approximate movement track at regular intervals (R package *crawl;* [45]). The intervals set in the CRAWL models were the same as the intervals at which the loggers were set to record geographic locations (i.e. 2 min). When penguins are underwater, GPS signal is lost, resulting in irregular time intervals between locations; the CRAWL method fits a movement model to estimate locations at regular time intervals rather than assuming linear movement between irregular location estimates [46]. Prior to further analysis, all GPS locations within a 2 km (penguins) or 15 km (flying birds) buffer around the island were removed to avoid an upward bias created by birds leaving and returning from foraging trips. This resulted in removal of all giant petrels that foraged exclusively within penguin and seal rookeries at the island.

### Identification of foraging behaviour along GPS tracks

Foraging activity along a seabird’s track is characterised by high sinuosity (i.e. frequent turning) and low flight speeds, and can be distinguished from direct and fast transit to and from the colony [47, 48].

To determine foraging locations along individual tracks of flying seabirds, Expectation Maximization binary Clustering (EMbC) was used (R package *EMbC;* [49]). This method uses an unsupervised clustering algorithm based on maximum-likelihood Gaussian mixture models that produces biologically interpretable behavioural classifications from flying seabird tracking data [50, 51]. Derived from the turning angle and speed between successive GPS locations, the EMbC determines four behavioural classification categories, obtained from the four combinations of high and low values of turning angle and speed. Locations categorized to have low speeds and high turning angles are considered as the birds ‘actively sitting’ and can be considered as the birds being in a behavioural foraging phase [50].

The EMbC did not perform well on the penguin data. However, for diving predators, speed is a useful proxy of foraging behaviour [52, 53], with slower speeds (reduced horizontal displacement) typically associated with increased foraging activity [54]. Speeds between successive GPS locations were calculated and speeds which were lower than a species average speed were identified as foraging locations.

### Stable isotope analysis

The high lipid contents within blood plasma, detected by a C:N mass ratio > 3.5 in tissues for aquatic animals [55], lead to artificially low δ^13^C values [56, 57]. This may lead to misinterpretation when inferring foraging locations of consumers from their δ^13^C tissue values. Mathematical normalization equations developed for a particular species or tissue may not be appropriate for another [55, 58], while chemical extraction methods may artificially increase δ^15^N values [59]. Therefore, δ^13^C and δ^15^N values were obtained from lipid extracted and raw plasma, respectively.

Blood plasma was dried at 50 °C for 48 h before being powdered using a mortar and pestle. Where possible, each plasma sample was divided into two aliquots: lipids were extracted from one half of the sample while the other half was analysed without lipid extraction. Lipids were removed by immersing powdered plasma in a 2:1 chloroform: methanol solution with a solvent volume three to five times greater than sample volume. Samples were then vortexed for 10 s every 10 min for 1 hr before being centrifuged for 5 min. The supernatant containing lipids was discarded, and samples dried at 50 °C overnight.

For small plasma samples where lipid extraction was not possible (two wandering, six grey-headed and one sooty albatross samples and two gentoo and five macaroni penguin samples), raw material was analysed so that true δ^15^N values could be obtained. To calculate lipid corrected δ^13^C values, species-specific mathematical normalization equations were developed using delipidated and raw plasma samples used in this study as well as others collected during the same breeding seasons but not presented here (Pistorius unpub. data), so that sample size could be increased. A species-specific normalized δ^13^C value was calculated using the difference of δ^13^C values (Δδ^13^C) and C:N ratios (ΔC:N) between non-delipidated plasma (δ^13^C and C:N) and delipidated plasma (δ^13^C_del_ and C:N_del_) for each individual (eq. 1 and eq. 2, respectively). A linear regression between Δδ^13^C and the ΔC:N ratio was then calculated, thus giving an intercept (c) and slope (m) for each species-specific equation (eq. 3). Eq. 3 was used to calculate Δδ^13^C for the samples that did not have a lipid free counterpart with ΔC:N for the raw samples calculated as the difference between their C:N and the average C:N of the delipidated samples. Finally, a corrected δ^13^C (δ^13^C_cor_) was calculated for samples without a lipid free counterpart using eq. 4. Species-specific equations (eq. 3) and sample sizes are given in Supplementary Material S2.

1 $$ \Delta {\updelta}^{13}\mathrm{C}={\updelta}^{13}{\mathrm{C}}_{\mathrm{del}}-{\updelta}^{13}\mathrm{C} $$

2 $$ \Delta \mathrm{C}:\mathrm{N}=\mathrm{C}:{\mathrm{N}}_{\mathrm{del}}-\mathrm{C}:\mathrm{N} $$

3 $$ \Delta{\delta}^{13}{\mathrm{C}} = \frac{\delta \mathrm{C}:\mathrm{N}+\mathrm{c}}{\mathrm{m}}$$

4 $$ {\updelta}^{13}{\mathrm{C}}_{\mathrm{cor}}={\updelta}^{13}\mathrm{C}+\Delta  {\updelta}^{13}\mathrm{C} $$

The isotopic values of carbon and nitrogen in aliquots (~ 0.4 mg) of homogenized delipidated and raw plasma samples were determined by combusting samples in a Flash 2000 organic elemental spectrometer via a Conflo IV gas control unit (Thermo Scientific, Germany). All samples were processed at the Stable Light Isotope Unit at the University of Cape Town, South Africa. Replicate measurements of internal laboratory standards indicated minimal standard deviations within and among runs (Merck gel: SD_δ13C_ = 0.2 ‰, SD_δ15N_ < 0.1 ‰; valine: SD_δ13C_ < 0.2 ‰, SD_δ15N_ = 0.1 ‰; seal bone: SD_δ13C_ < 0.2 ‰, SD_δ15N_ < 0.1 ‰). All in-house standards were calibrated against International Atomic Energy Agency standards. Results are presented in the usual δ notation relative to Vienna PeeDee Belemnite and atmospheric N_2_ for δ^13^C and δ^15^N values, respectively.

### Estimation of Isoscapes

Blood plasma has a half-life of a few days [22]. It was therefore assumed that the δ^13^C and δ^15^N values of the seabirds’ blood plasma reflected approximately 7 days bioaccumulation of isotope value of prey prior to sampling. The isotope value of a seabird’s tissue is a moving temporal window of what it has ingested [22, 35]. Thus, a geographic location that would represent an individual’s single δ^13^C and δ^15^N plasma values was estimated by identifying cells where the individual was likely foraging (see Identification of foraging behaviour along GPS tracks) and weighting each of these cells by the proportion of time spent foraging within a given cell. Calculating the mean of these weighted positions resulted in a time-integrated weighted mean foraging location for each individual. In the case of brooding albatrosses (grey-headed and sooty albatrosses only) and penguins, where more than one foraging trip was recorded, multiple trips were included in further analyses if they did not exceed 7 days prior to blood collection.

The proportion of time spent per cell was calculated (R package *trip;* [42]). Since the path length and duration of albatrosses and giant petrels was much greater than that of the penguins, two different grid sizes were used for flying and diving seabirds when calculating time spent per cell. The grid cell size was estimated by taking the average distance that the flying or diving species travelled in 2 h (as location interval for flying birds was 1 hr) and rounding up to the nearest 0.05°. This resulted in grid cell sizes of 0.5° for flying birds and 0.05° for diving birds.

Possible geographic gradients were investigated using Pearson’s correlation to identify relationships between δ^13^C and δ^15^N values of plasma and mean foraging latitude and longitude (in flying species) or distance to coastline (in diving species). As all penguin species only moved within ~ 0.5° of latitude, their blood plasma isotope values are unlikely to be affected by latitude and distance to coastline was thus used to investigate a possible inshore to offshore gradient. To visualize δ^13^C and δ^15^N isoscapes, species-specific geostatistical models were used to interpolate δ^13^C and δ^15^N values among the mean foraging locations. Ordinary kriging is a spatial interpolation method which relies on the notion of autocorrelation as a function of distance [60] and has been shown to perform well when estimating isoscapes from point locations [61, 62]. Thus, ordinary kriging (R package *gstat;* [63]) was used, with best fit variograms identified (R package *automap;* [64]). Interpolations were made onto the same grid used to calculate time-spent per cell per individual, with extent of the grid being a convex hull around a species mean foraging locations with a 0.5° or 0.05° buffer for flying species and penguins, respectively. Isoscapes estimated for each species were then subtracted from one another to allow for comparison among the isocapes produced for each species, following the methods used by St John Glew et al. [61].

The mean foraging location and plasma isotope values from all species within a guild (i.e. flying birds and penguins) were then combined to produce guild-specific δ^13^C and δ^15^N isoscapes. To account for the different number of individuals per species, a bootstrap approach was used. Whereby, 22 and 9 individuals (the lowest number of individuals per flying and penguin species, Table 1) were randomly sampled 1000 times from each of the flying and penguin species, respectively, and used to produce new δ^13^C and δ^15^N isoscapes. The mean of these isoscapes was then calculated to present guild-specific isoscapes which consider different number of individuals per species.

To investigate possible δ^13^C and δ^15^N values characteristic of main fronts and water masses within the Southern Ocean, isotopic values were extracted within one degree along the sub-tropical, sub-Antarctic and Antarctic polar fronts as well as for sub-tropical, sub-Antarctic, polar frontal and Antarctic water zones. Front positions were estimated from satellite derived sea surface height (Ssalto/Duacs produced and distributed by the Copernicus Marine Environment Monitoring Service http://marine.copernicus.eu) averaged over December of the 3 years of the study (2015–2017). Following Swart et al. [65], the fronts were identified from the following sea surface heights: sub-tropical: 0.92 m, sub-Antarctic: 0.03 m and Antarctic polar: − 0.48 m. The four water zones identified were considered as the following: the subtropical (north of the sub-tropical front), the sub-Antarctic (between the sub-tropical and sub-Antarctic fronts), the polar frontal zone (between the sub-Antarctic and Antarctic polar fronts) and the Antarctic (south of the Antarctic polar front). In addition, the δ^13^C and δ^15^N values of the Antarctic polar (51°S) and sub-tropical fronts (42°S) were back calculated using estimated regression equations following Jaeger et al. [14].

All data analyses were performed using R version 3.6.3 [66]. All values are reported as mean ± standard deviation and significance is specified as *p* ≤ 0.05.

## Results

Over the duration of the study, 176 birds were tracked, and their blood plasma analysed for δ^13^C and δ^15^N values (Table 1).

### Influence of seabird species on the detection of large-scale spatial variability in δ^13^C and δ^15^N isoscapes

**Fig. 1:**
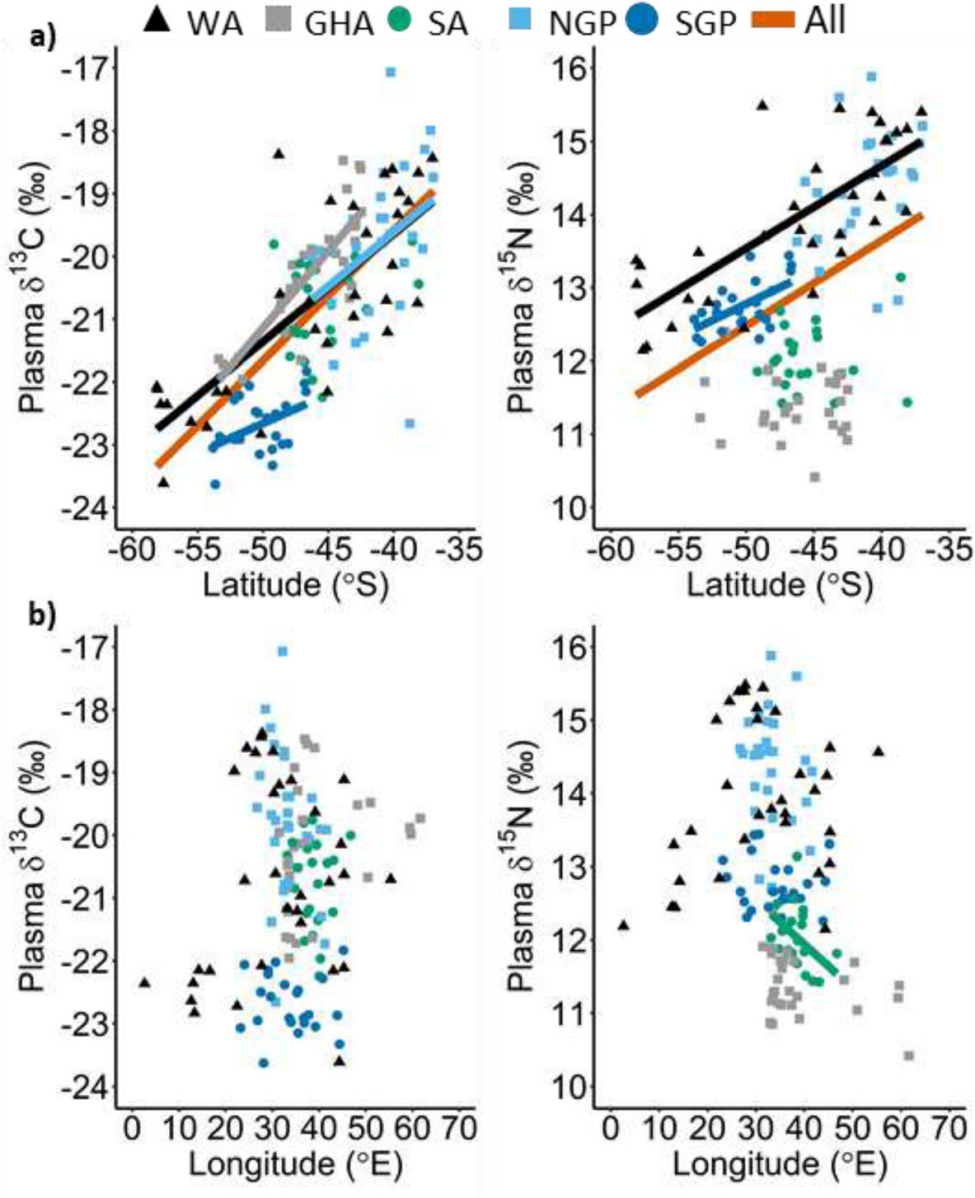
Plasma δ^13^C (left) and δ^15^N (right) values of wandering (WA), grey-headed (GHA) and sooty (SA) albatrosses, northern (NGP) and southern (SGP) giant petrels versus their mean foraging a) latitudes and b) longitudes calculated from GPS tracks. Only significant correlations are shown (see Table 2)

**Fig. 2:**
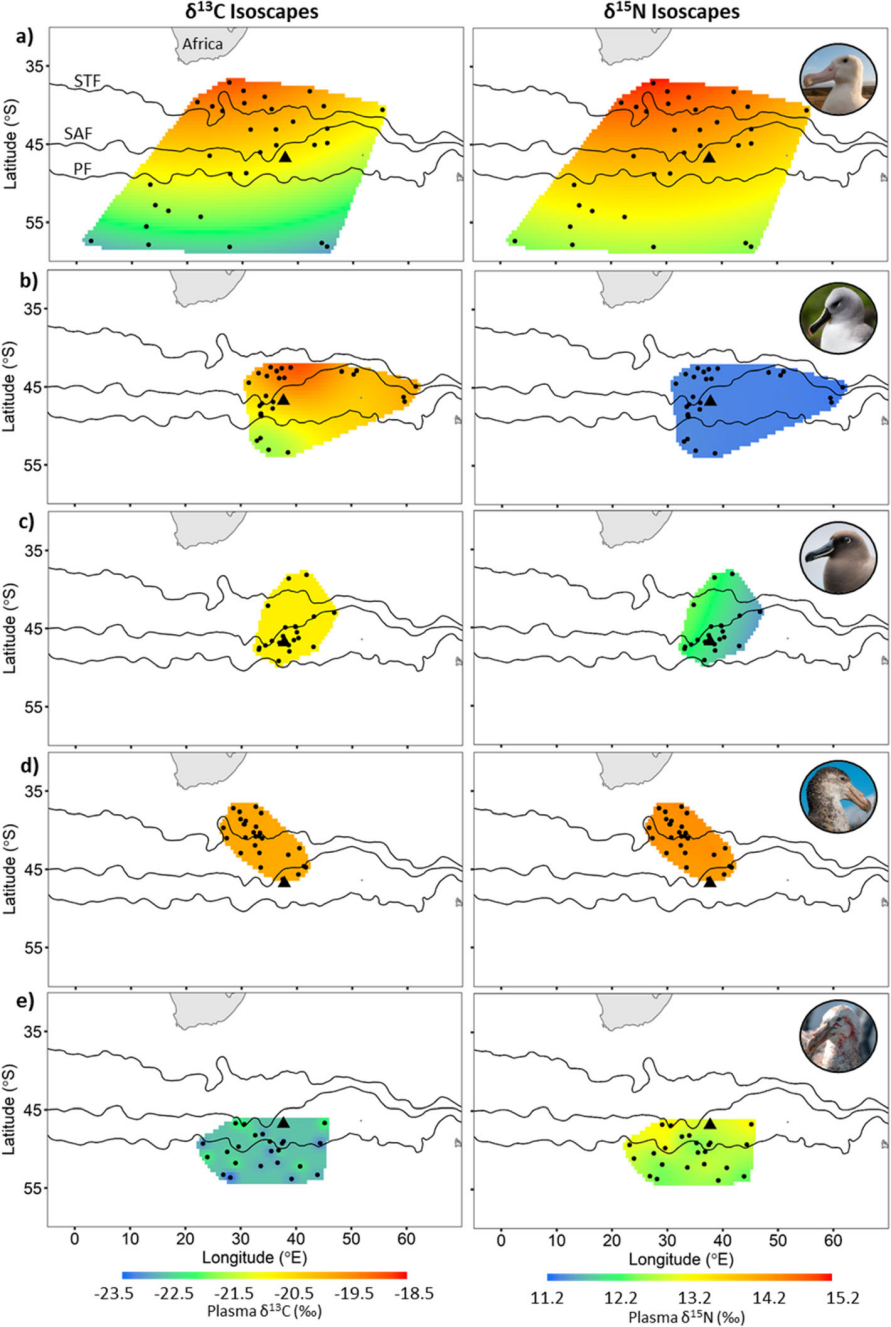
Plasma δ^13^C (left) and δ^15^N (right) isoscapes of **a**) wandering, **b**) grey-headed and **c**) sooty albatrosses, and **d**) northern and **e**) southern giant petrels breeding at Marion Island, Prince Edward Archipelago (black triangle) interpolated using ordinary kriging from the isotopic values of the respective birds’ plasma which were simultaneously tracked with GPS data loggers. Black points represent an individual’s mean foraging location. Positions of the sub-tropical (STF), sub-Antarctic (SAF) and Antarctic polar (PF) fronts are indicated

**Fig. 3:**
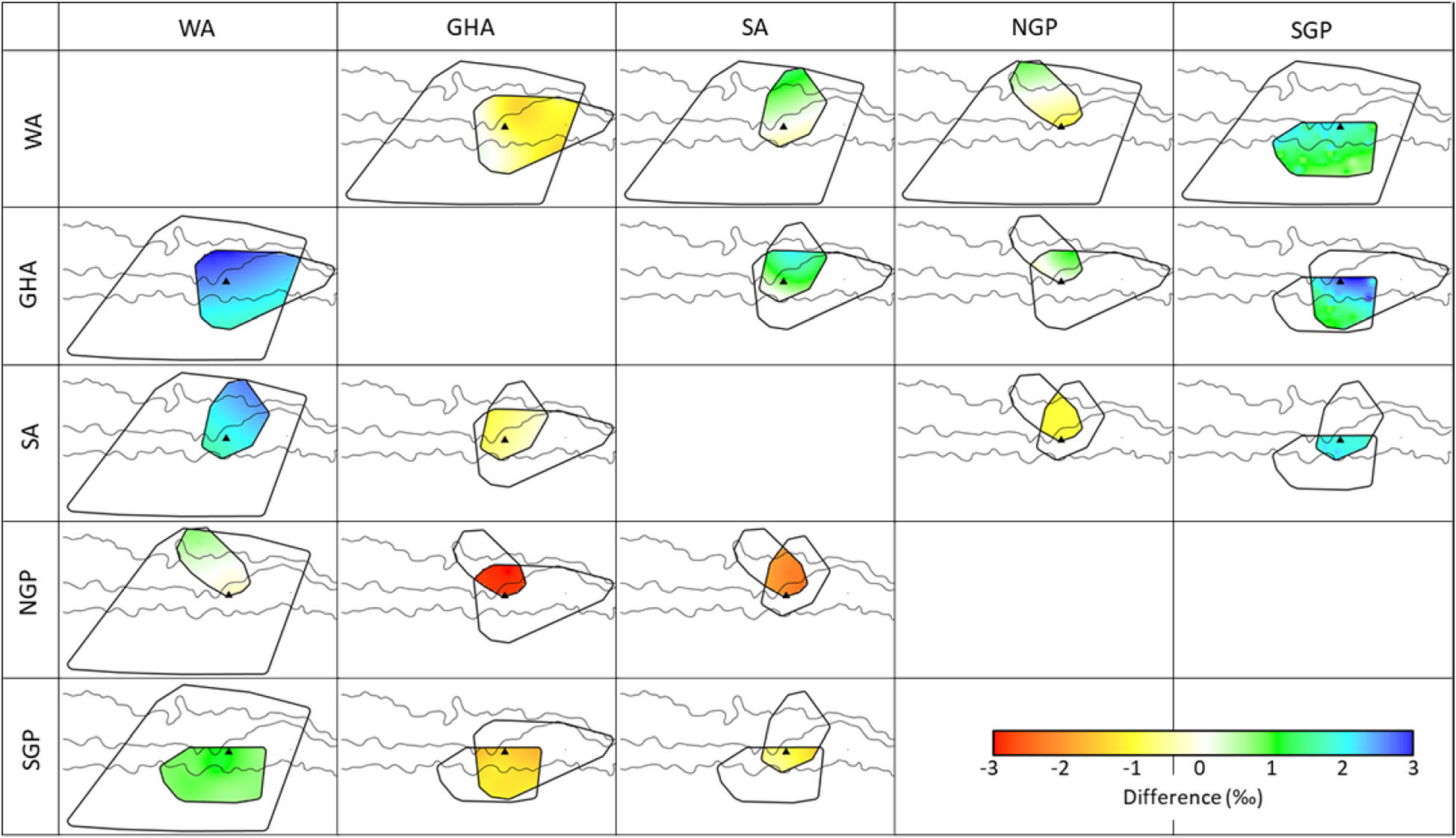
Difference between wandering (WA), grey-headed (GHA) and sooty (SA) albatross as well as northern (NGP) and southern (SGP) giant petrel plasma δ^13^C (top right diagonal) and δ^15^N (bottom left diagonal) isoscapes. Differences were calculated by subtracting the isoscape of the species indicated on the vertical axis from the species indicated on the horizontal axis. Due to the small overlap between isoscapes of the giant petrels their comparison is not shown

The δ^13^C blood plasma values of all flying seabird species were positively correlated to their mean foraging latitude and significantly so with the exception of the sooty albatross (Table 2, Fig. 1). The correlation between the mean foraging latitude and δ^13^C plasma values of the grey-headed albatross was the strongest (R = 0.83) followed closely by the wandering albatross (R = 0.78) and southern (R = 0.37) and northern (R = 0.37) giant petrels. On the other hand, none of the species δ^13^C plasma values and mean foraging longitudes were significantly correlated (Table 2; Fig. 2). These findings were apparent in δ^13^C isoscapes interpolated for the wandering and grey-headed albatrosses where a clear north to south gradient of δ^13^C values can be seen (Fig. 2a and b). The comparison between the wandering and grey-headed albatross isoscapes showed that the grey-headed albatross δ^13^C isoscape was ~ 1‰ greater than that of wandering albatross within the area of overlap, that is north of the sub-tropical front (Fig. 3). This was reflected with the species regression equations (Fig. 1) with greater δ^13^C values being predicted for the grey-headed albatross than the wandering albatross at similar latitudes. Although the correlation between mean foraging latitude and δ^13^C values of northern and southern giant petrels were significant, the correlation was not as apparent in the interpolated surface (Fig. 2d and e). No pattern could be discerned from the interpolated surface resulting from the sooty albatross’s mean foraging locations and δ^13^C values (Fig. 2c).

**Table 2:** Pearson’s correlation coefficient (R), *p*-value (P) and formula resulting from a Pearson’s correlation between plasma δ^13^C and δ^15^N values of albatrosses and giant petrels versus latitude (lat) and longitude (lon) of their mean foraging locations calculated from GPS tracks. ^a^ indicates a significant correlation

	δ^13^C vs Latitude			δ^13^C vs Longitude			δ^15^N vs Latitude			δ^15^N vs Longitude		
Species	R	P	Formula	R	P	Formula	R	P	Formula	R	P	Formula
**All species combined**												
	0.73	< 0.01^a^	δ^13^C = 0.21 (lat) - 11.25	0.12	0.17	δ^13^C = 0.02 (lon) - 21.48	0.44	< 0.01^a^	δ^15^N = 0.12 (lat) + 18.29	0.24	0.19	δ^15^N = 0.02 (lon) + 13.33
**Albatrosses**												
Wandering	0.78	< 0.01^a^	δ^13^C = 0.17 (lat) - 12.76	0.21	0.25	δ^13^C = 0.03 (lon) - 21.55	0.76	< 0.01^a^	δ^15^N = 0.11 (lat) + 19.19	0.24	0.19	δ^15^N = 0.02 (lon) + 13.33
Grey-headed	0.83	< 0.01^a^	δ^13^C = 0.24 (lat) - 8.81	0.27	0.17	δ^13^C = 0.03 (lon) - 21.40	− 0.11	0.57	δ^15^N = 0.01 (lat) + 10.74	−0.36	0.06	δ^15^N = 0.02 (lon) + 12.09
Sooty	0.25	0.27	δ^13^C = 0.06 (lat) - 18.00	−0.02	0.93	δ^13^C = 0.004 (lon) - 20.63	0.09	0.69	δ^15^N = 0.01 (lat) + 12.67	− 0.45	0.03^a^	δ^15^N = 0.06 (lon) + 14.35
**Giant petrels**												
Northern	0.37	0.05^a^	δ^13^C = 0.17 (lat) - 12.71	−0.31	0.11	δ^13^C = 0.09 (lon) - 16.79	0.29	0.14	δ^15^N = 0.08 (lat) + 17.81	− 0.16	0.43	δ^15^N = 0.03 (lon) + 15.33
Southern	0.47	0.02^a^	δ^13^C = 0.10 (lat) - 17.73	− 0.07	0.77	δ^13^C = 0.01 (lon) - 22.52	0.58	< 0.01^a^	δ^15^N = 0.09 (lat) + 17.43	−0.12	0.58	δ^15^N = 0.01 (lon) + 12.99

The δ^15^N plasma values of two of the five flying species were significantly and positively correlated to mean foraging latitude, namely the wandering albatross and southern giant petrel (Table 2, Fig. 1). Contrastingly, the δ^15^N plasma values of sooty albatrosses, which were not significantly correlated to mean foraging latitude (Table 2), were significantly correlated to mean foraging longitudes (Table 2, Fig. 1b). The resulting interpolated surface of mean foraging location and δ^15^N plasma values of the wandering albatross and southern giant petrel showed clear gradients of δ^15^N values from north to south (Fig. 2a and e). Although, the resulting wandering albatross δ^15^N isoscape was ~ 1‰ greater than that of the southern giant petrel across the interpolated surface (Fig. 3). On the contrary, the interpolated surfaces resulting from the mean foraging locations and δ^15^N plasma values of sooty albatrosses showed a gradient from west to east (Fig. 2c).

**Fig. 4:**
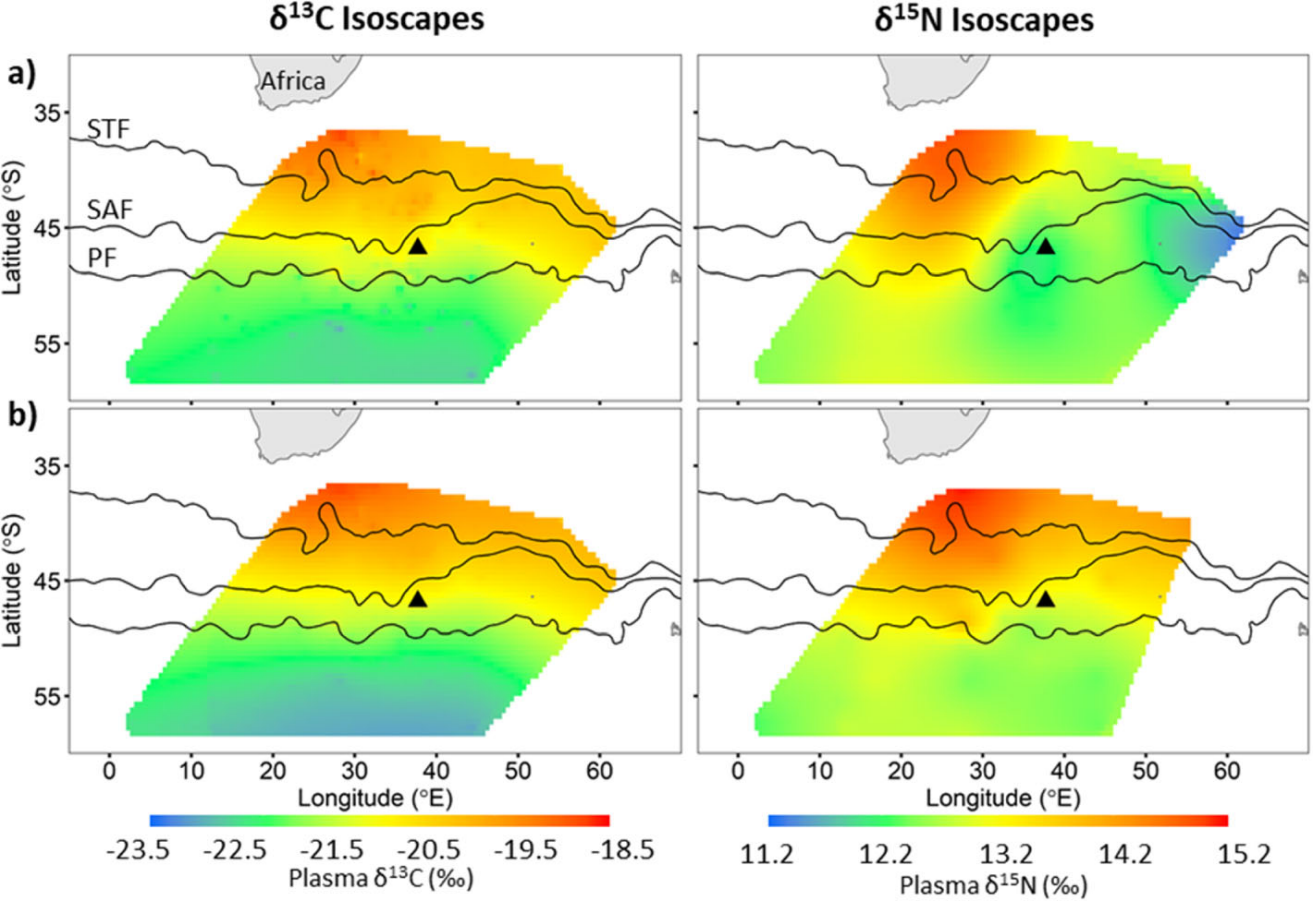
**a** Plasma δ^13^C (left) and δ^15^N (right) isoscapes calculated as the mean of 1000 bootstrapped surfaces interpolated using ordinary kriging which randomly sampled twenty-two individuals from all five flying species to account for different numbers of individuals per species. **b** Isoscapes, using the same bootstrap technique, modelled without the species which did not exhibit a significant latitudinal gradient (i.e. sooty albatrosses were removed from the δ^13^C isoscape and sooty and grey-headed albatrosses and northern giant petrels were removed from the δ^15^N isoscape). Position of the sub-tropical (STF), sub-Antarctic (SAF) and Antarctic polar (PF) fronts and Marion Island (black triangle) are indicated

The δ^13^C and δ^15^N plasma values of all flying seabirds combined were significantly correlated with mean foraging latitude (R = 0.73, *p* < 0.01 and R = 0.44, p < 0.01, respectively; Table 2, Fig. 1a, b) but not to mean foraging longitude (R = 0.12, *p* = 0.17 and R = 0.24, *p* = 0.19, respectively). The resulting isoscapes, interpolated from mean foraging locations and plasma δ^13^C values of all flying seabirds showed a clear south to north gradient in δ^13^C values (Fig. 4a). As δ^15^N plasma values of the flying seabirds were not as strongly correlated to latitude (R = 0.44), the resulting δ^15^N isoscape did not result in a clear south – north gradient, relative to the δ^13^C isoscape (Fig. 4a). However, there was also an increase in δ^15^N values from south – north with the higher δ^15^N values in the north-west area of the isoscape compared to those of the north-east (Fig. 4a).

As a post-hoc analysis, all species for which δ^13^C (sooty albatrosses) and δ^15^N (grey-headed and sooty albatrosses and northern giant petrel) plasma values were not significantly related to their mean foraging latitude were removed and the remaining isoscapes were re-interpolated (Fig. 4b). This resulted in a similar δ^13^C isoscape to the previously estimated δ^13^C isoscape (Fig. 4a). However, the resulting δ^15^N isoscape now showed a clear north to south gradient (Fig. 4b).

### δ^13^C and δ^15^N isotopic values of major fronts and water zones within seabird species isoscapes

**Fig. 5:**
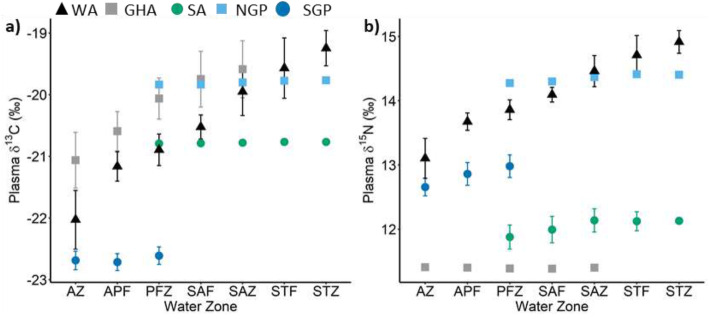
The means and standard deviations of (**a**) δ^13^C and (**b**) δ^15^N values estimated for seven water zones and fronts: Antarctic zone (AZ), Antarctic polar front (APF), polar frontal zone (PFZ), sub-Antarctic front (SAF), sub-Antarctic zone (SAZ), sub-tropical front (STF) and sub-tropical zone (STZ) from the species-specific isoscapes presented in Fig. 2: wandering (WA), grey-headed (GHA) and sooty (SA) albatrosses, northern (NGP) and southern (SGP) giant petrels

From each species-specific isoscape derived from flying seabirds (Fig. 2), mean isotopic values of the sub-tropical, sub-Antarctic and Antarctic polar fronts as well as four geographical water zones (sub-tropical, sub-Antarctic, polar frontal and Antarctic water zones) were estimated (Fig. 5, Supplementary Material S3). There were noticeable increases in δ^13^C values at the Antarctic polar and sub-Antarctic fronts within isoscapes interpolated for wandering and grey-headed albatrosses as well as at the sub-tropical and sub-Antarctic fronts compared to surrounding water zones (Fig. 5, Supplementary Material S3). With regards to δ^15^N values, there were noticeable decreases for water zones from north to south within the interpolated areas for the wandering albatross and southern giant petrel, whereas the remaining species (sooty and grey-headed albatrosses and northern giant petrel) showed no clear trend (Fig. 5, Supplementary Material S3). Following Jaeger et al. [14], regression equations calculated here (Table 2) were also used to back calculate the δ^13^C and δ^15^N values in plasma of the Antarctic polar and sub-tropical fronts at 51°S and 42°S (Table 3). The resulting δ^13^C values varied much less at both the Antarctic polar (− 21.6 ± 0.6 ‰) and sub-tropical fronts (− 20.3 ± 0.9 ‰) than the resulting δ^15^N values (12.5 ± 1.4 ‰ and 13.1 ± 1.8 ‰; respectively).

**Table 3:** Plasma δ^13^C and δ^15^N values calculated using species-specific regression equations (Table 2) for the Antarctic polar (PF, 51°S) and sub-tropical (STF, 42°S) fronts following the methods of Jaeger et al. [14]

Species	δ^13^C (‰)		δ^15^N (‰)	
	PF	STF	PF	STF
**Albatrosses**				
Wandering	−21.4	− 19.9	13.6	14.6
Grey-headed	−21.6	−19.4	10.3	10.4
Sooty	−21.1	− 20.5	12.2	12.3
**Giant petrels**				
Northern	−21.4	−19.9	13.7	14.5
Southern	−22.6	−21.8	12.7	13.5

### Fine scale spatial variability in δ^13^C and δ^15^N isoscapes estimated from seabird tissues

**Fig. 6:**
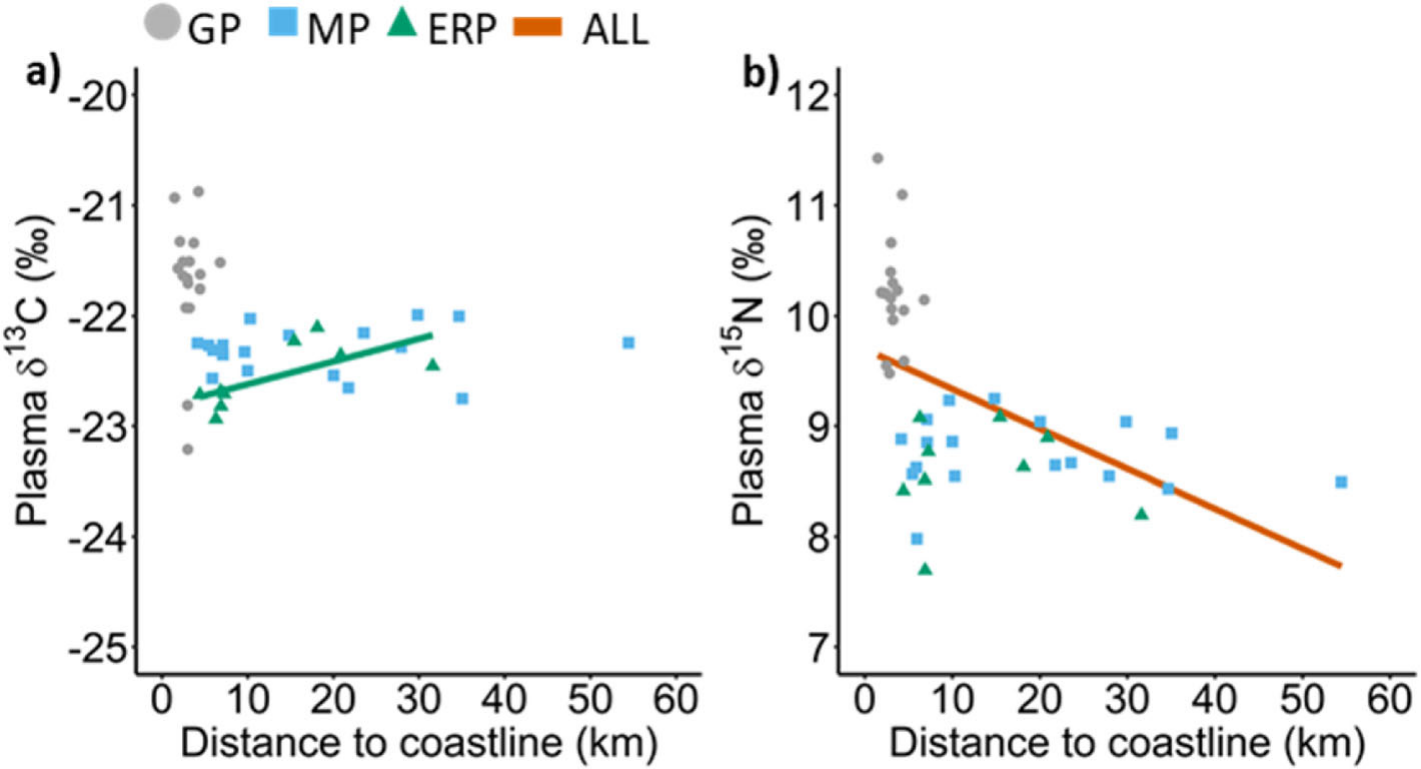
Plasma (**a**) δ^13^C and (**b**) δ^15^N values of gentoo (GP), macaroni (MP) and eastern rockhopper (ERP) penguins and all penguin species (ALL) combined versus the distance between their mean foraging locations and the coastline of Marion Island. Only significant correlations are shown

**Fig. 7:**
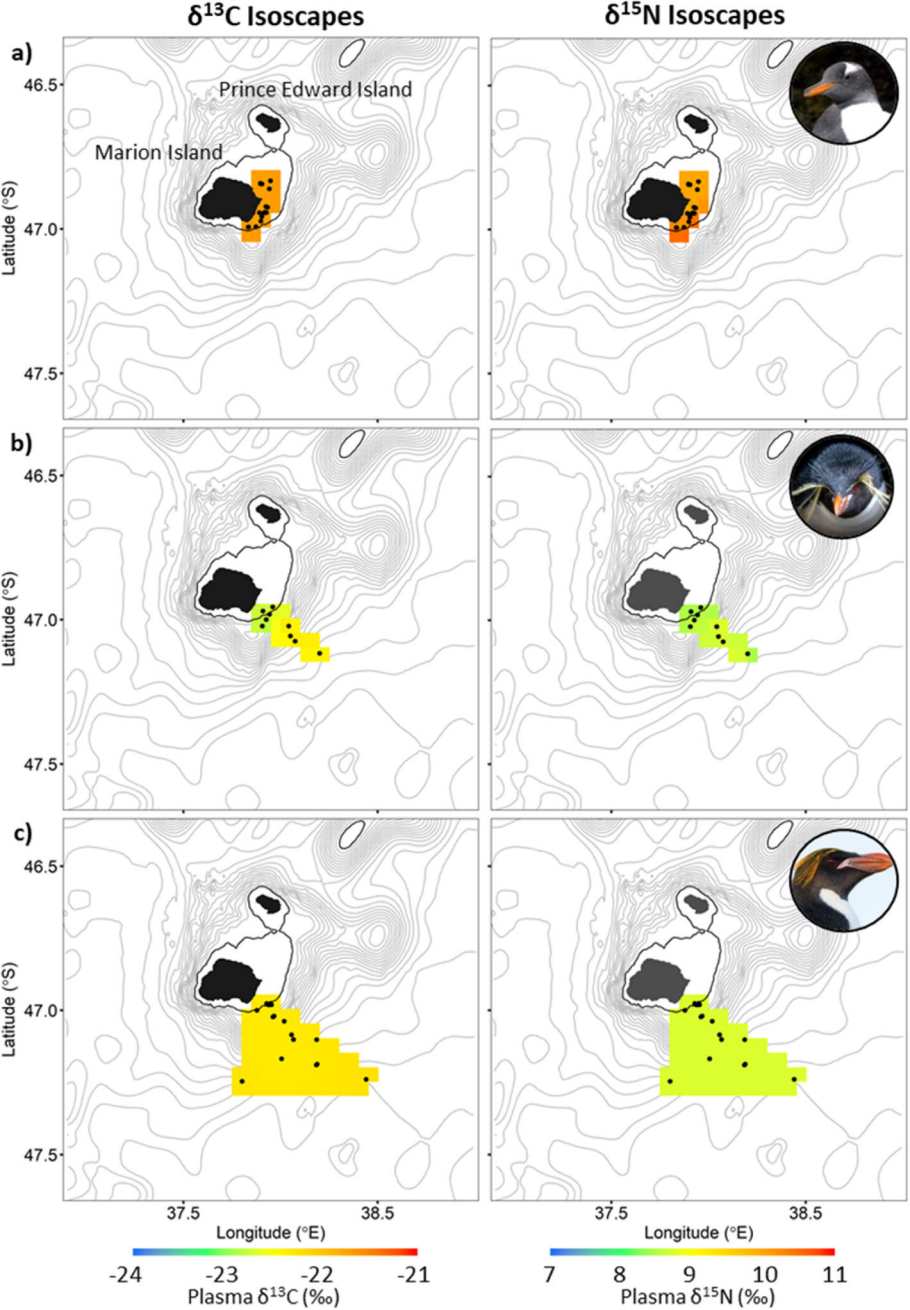
Plasma δ^13^C (left) and δ^15^N (right) isoscapes of **a**) gentoo, **b**) eastern rockhopper and **c**) macaroni penguins interpolated using ordinary kriging from the isotopic composition of the respective birds’ plasmas which were simultaneously tracked with GPS data loggers. Points represent mean foraging locations. Isobaths (grey) are shown at 200 m intervals with 200 m isobath in black

**Fig. 8:**
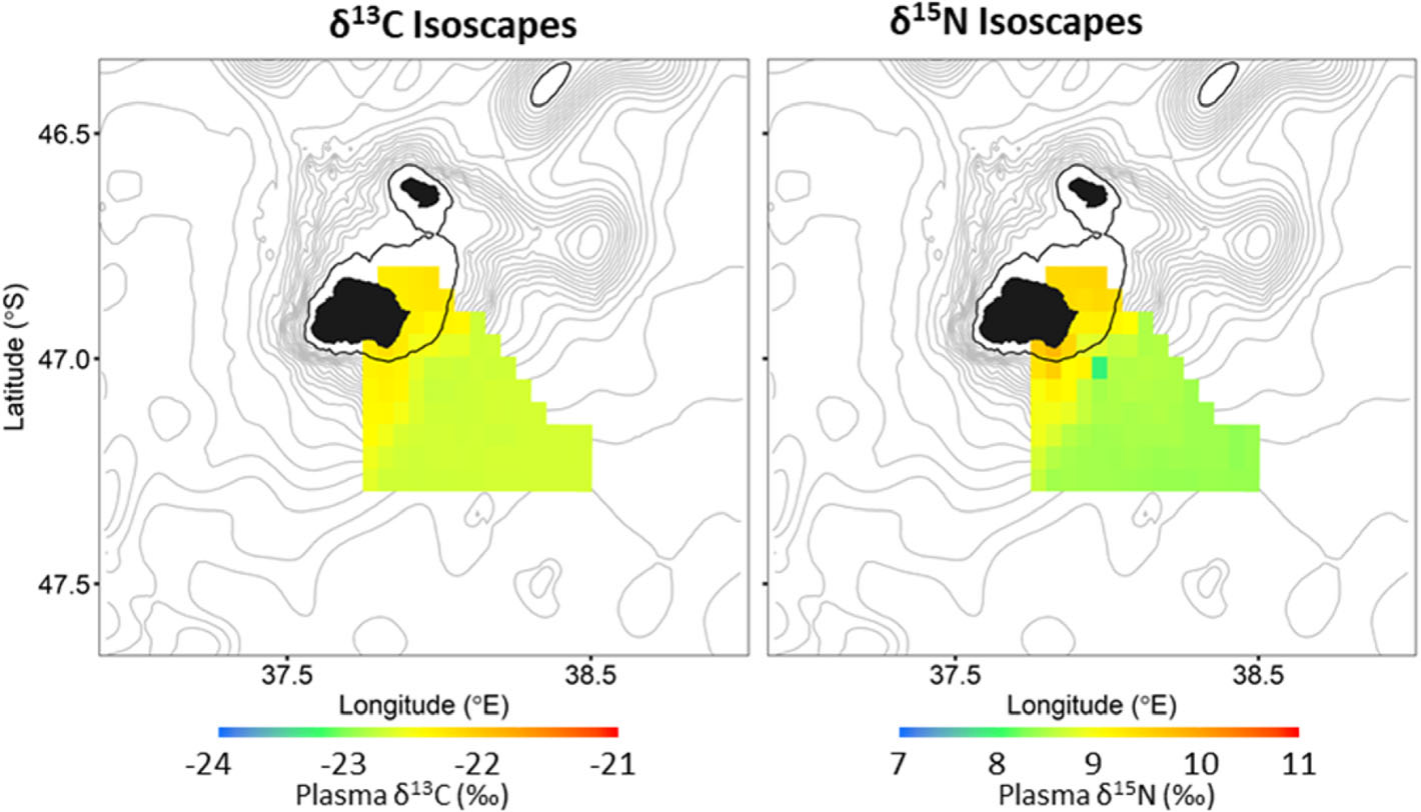
Plasma δ^13^C (left) and δ^15^N (right) isoscapes of three penguin species breeding at Marion Island, Prince Edward Archipelago calculated as the mean of 1000 bootstrapped surfaces interpolated using ordinary kriging which randomly sampled 9 individuals from each penguin species to account for different number of individuals per species. Isobaths (grey) are shown at 200 m intervals with 200 m isobath in black

No significant correlations were found between the distance to the coast and mean foraging location of the penguin species and their corresponding δ^13^C and δ^15^N plasma values (Table 4, Figs. 6 and 7). Except for the δ^13^C values of the eastern rockhopper penguin. However, the δ^15^N plasma values of all penguins combined was significantly correlated with the distance between their mean foraging locations and the coastline of Marion Island (Table 4; Figs. 6 and 8). Furthermore, both δ^13^C and δ^15^N penguin-specific isoscapes showed a clear gradient of higher values closer to the island, in waters shallower than 200 m, to lower values further away, in deeper waters (Fig. 8).

**Table 4:** Correlation coefficient (R), p-value (P) and formula resulting from a Pearson’s correlation between δ^13^C and δ^15^N plasma values of individual penguin species as well as all species combined versus the distance between their mean foraging locations and the coastline of Marion Island (distTOcoast). ^a^ indicates a significant correlation

	δ^13^C vs distTOcoast			δ^15^N vs distTOcoast		
	R	P	Formula	R	P	Formula
**All species combined**						
	−0.26	0.08	δ^13^C = 0.01 (distTOcoast) - 21.99	−0.49	< 0.01^a^	δ^15^N = 0.03 (distTOcoast) + 9.69
**Penguins**						
Gentoo	0.03	0.92	δ^13^C = 0.01 (distTOcoast) - 21.74	−0.10	0.71	δ^15^N = 0.03 (distTOcoast) + 10.32
Macaroni	0.08	0.77	δ^13^C = 0.001 (distTOcoast) - 22.33	−0.10	0.70	δ^15^N = 0.002 (distTOcoast) + 8.80
Rockhopper	0.66	0.05^a^	δ^13^C = 0.02 (distTOcoast) - 22.82	−0.13	0.74	δ^15^N = 0.01 (distTOcoast) + 8.67

## Discussion

By combining fine-scale tracking data and stable isotope analysis, we present a critical assessment of concurrent δ^13^C and δ^15^N gradients across a marine predator assemblage. This study revealed latitudinal spatial gradients in both δ^13^C and δ^15^N plasma values for far-ranging seabirds (i.e. albatrosses and giant petrels) as well as inshore/offshore gradients for near-ranging seabirds (penguins). Despite demonstrating clear potential for using seabird tissue samples for retrospective geolocation, the inter-specific differences in δ^13^C and δ^15^N isoscapes cautions against using non-species-specific isoscapes for studying marine predator foraging distributions.

### Influence of seabird species on the detection of large-scale spatial variability in δ^13^C isoscapes

Strong positive correlations were found between the mean foraging latitudes and δ^13^C plasma values of two out of the five flying seabird species, namely the wandering and grey-headed albatrosses. Differences between these two species isoscapes (Figs. 2, 3 and 4) as well as the lack of gradients found within the δ^13^C isoscapes of the remaining species is likely due to differences in diet [67] and the use of different oceanographic features as foraging grounds [68–70].

1. **Influence of diet on a species-specific δ**^**13**^**C isoscape**

It was difficult to discern any spatial gradient within the δ^13^C isoscapes of the northern and southern giant petrels and the sooty albatross. This lack of a strong latitudinal gradient in the δ^13^C plasma values of these species may be attributed to their more unpredictable diet. For example, at Marion Island the diet of all three species may contain large proportions of seabird and marine mammal carrion [71, 72]. The relatively narrow range of both the southern giant petrel and sooty albatross δ^13^C plasma values (Table 1) may suggest that either they preyed on the same seabird carrion or different carrion that had been foraging in similar areas. Without concurrent stomach content sample analysis this is largely speculation, however the lack of spatial gradient found for the δ^13^C and δ^15^N plasma values of the southern giant petrel and sooty albatross does indicate that caution is needed when using non-species specific isoscapes to infer foraging locations of seabirds from their δ^13^C tissue values.

2. **Influence of foraging habitat on a species-specific δ**^**13**^**C isoscape**

Grey-headed albatrosses from Marion Island remain largely south of the sub-tropical front (this study [68]), whereas wandering albatross (this study [69]) frequently move north of the sub-tropical front. However, both species are likely foraging within and around biologically productive eddies that result from interactions between the Agulhas Return Current, sub-tropical and sub-Antarctic fronts [73], or within the fronts themselves [68, 74, 75]. As δ^13^C values at the base of marine food webs have been shown to significantly correlate with primary productivity and nutrient availability [25, 27], birds foraging around or within mesoscale eddies originating from the same oceanographic feature are likely to have elevated δ^13^C tissue values compared to species that forage outside of fronts and eddies. Thus, the higher δ^13^C plasma values at lower latitudes for the grey-headed albatross compared to other species (Figs. 3 and 4) may result from the grey-headed albatross preferentially foraging in highly productive eddies [68]. This is further supported by the similar δ^13^C values found for the wandering and grey-headed albatrosses and northern giant petrel within the sub-tropical front as well as within the sub-Antarctic and sub-tropical water zones (Fig. 5; Supplementary Material S3).

3. **Influence of baseline δ**^**13**^**C gradient on a species-specific isoscape**

The δ^13^C combined species isoscape as well as that of the wandering albatross closely aligns with previously published isoscapes for this region, which used similar methods but only with a single species (wandering albatross; [14]), used whole blood of close-ranging seabirds [17] or baseline values (e.g. [16]). Previous studies, which have used a coupled physics-biochemistry model [16] or satellite tracking coupled with predator tissue values [14] to produce δ^13^C isoscapes for the Indian sector of the Southern Ocean, have shown uniform south-north latitudinal gradient in δ^13^C values. In agreement, presented here is a large scale south-north δ^13^C isoscape, albeit with slightly non-uniform latitudinal gradient in δ^13^C values. The isoscape presented by Magozzi et al. [16] had a greater range of δ^13^C values between southern Africa and Antarctic (− 32 to − 16 ‰) compared to our isoscapes (− 23 to − 18 ‰). Our predator-based isoscape may differ to that of Magozzi et al.’s [16] for two reasons. Firstly they modelled the distribution of δ^13^C values of phytoplankton and not seabirds. Secondly, Magozzi et al. [16] showed that in the Indian sector of the Southern Ocean, δ^13^C values of phytoplankton become increasingly more negative with the transition of summer to winter months. Therefore, the narrower range of δ^13^C values obtained in our δ^13^C isoscape can be related to the enrichment of δ^13^C values due to fractionation through multiple trophic levels from phytoplankton to seabirds [55, 76], as well as the temporal integration of spatially and temporally variable δ^13^C phytoplankton values [77].

4. **Other influences on seabirds δ**^**13**^**C isoscapes**

The isoscape map and regression equation of Jaeger et al. [14] for the wandering albatross tracked from Crozet Archipelago (using similar methods used here), revealed higher δ^13^C values with a steeper δ^13^C gradient (range: − 25.0 to − 19.1 ‰) than we found for Marion Island wandering albatrosses (range: − 23.6 to − 18.4 ‰) over similar latitudes (~ 38–58°S). This could be an artefact of differences in diet between the two populations of wandering albatrosses [67]. However, the mean δ^15^N plasma value of the wandering albatross in our study (mean: 14.0 ‰; range: 12.1 to 15.5‰) and Jaeger et al.’s [14] study (14.0 ‰; 11.2 to 15.8 ‰) was the same, indicating that individuals from the two populations were foraging at similar trophic levels. Rather, a difference in the methods used to remove lipids from plasma between the two studies (cyclohexane [14] versus a 2:1 chloroform to methanol mix (our study)) may explain the slightly more negative δ^13^C values reported by Jaeger et al. [14]. This is supported by the fact that Jaeger et al. [14] reported a higher C:N ratio (4.1) than we found for the wandering albatross plasma (3.5), which indicates that Jaeger et al. [14]‘s samples had a higher percentage of lipids than ours. Because lipids can lead to artificially low δ^13^C values [56, 57], this may explain their lower δ^13^C plasma values.

These finding once again highlight the importance of removing or accounting for lipids when inferring the retrospective foraging location of seabirds from their δ^13^C tissue values [56, 78]. Especially, since during different life-history stages, seabirds may undergo differential levels of nutritional stress such as periods of fasting, which may result in the increase of lipids circulating in the bloodstream [79]. As previously mentioned, this can lead to artificially low δ^13^C values [56, 57]. However, as δ^13^C blood values are not (or little) otherwise affected when seabirds undergo nutritional stress [79], we believe the δ^13^C isoscapes produced here are transferable across life-history stages of seabirds.

### Influence of seabird species on the detection of large-scale spatial variability in δ^15^N isoscapes

This study revealed a south to north gradient of the wandering albatross and southern giant petrel δ^15^N plasma values whereas δ^15^N plasma values of the grey-headed and sooty albatrosses and northern giant petrel lacked a south to north gradient. However, the δ^15^N plasma values of the sooty albatross did present a relatively weak west to east gradient. The δ^15^N value of predators’ tissues is a combination of their trophic position and δ^15^N values of organisms at the base of the food chain [55, 76]. Therefore, as presented by Jaeger et al. [14], there are two possible and non-exclusive hypotheses to explain systematic spatial variability in seabird δ^15^N tissue values: firstly, a dietary shift of the predators throughout the predator’s distributional range and secondly a change in the baseline nitrogen values.

1. **Influence of diet on a species-specific δ**^**15**^**N isoscape**

Jaeger et al. [14] provided evidence for the first hypothesis by using previous studies of the wandering albatross diet to show that, although the wandering albatross breeding at Crozet Archipelago feed on squid across their range, Antarctic squid species feed at lower trophic levels than sub-Antarctic and tropical squid species [24], which may result in the latitudinal gradient of δ^15^N plasma values. The same data is not available for the wandering albatross breeding at Marion Island, but it is reasonable to assume that this is also a factor driving the spatial gradient of the wandering albatross δ^15^N plasma values within this study.

2. **Influence of baseline δ**^**15**^**N gradient on a species-specific δ**^**15**^**N isoscape**

Although diet is undoubtedly an important contributing factor to the δ^15^N plasma values of the birds within this study, this study provides evidence towards Jaeger et al. [14]‘s second hypothesis: baseline δ^15^N values influence the latitudinal gradient in δ^15^N plasma values of seabirds. Evidence supporting this argument is that the wandering albatross and southern giant petrel had similar regression slopes between mean foraging latitudes and δ^15^N plasma values. The difference between these two species δ^15^N isoscapes was homogenously 1 ‰ across overlapping areas (Fig. 3), which may reflect difference in their diets [67]. Furthermore, the wandering albatross and southern giant petrel δ^15^N isoscapes as well as the δ^15^N isoscape resulting from the combination of their data (Fig. 4b) resembled the latitudinal gradients previously found for δ^15^N values across the Indian sector of the Southern Ocean for seabirds (wandering albatross: [14]) and baseline values [20]. The values within these δ^15^N isoscapes ranged between ~ 12 ‰ in the south and ~ 15 ‰ in the north, closely matching the ~ 2 ‰ modelled gradient in baseline δ^15^N values between Antarctica and the coastline of southern Africa [20]. This indicates that these spatially variable baseline values propagate up the food chain and can be observed in the δ^15^N tissue values of the seabirds. These are important findings for the use of stable isotope analysis for the retrospective geolocation of marine consumers. Previously, δ^13^C values of predator tissues have been used for retrospective geolocation, however, agreement among trends found within δ^15^N isoscapes in this study and two others [14, 20] provide evidence toward the use of δ^15^N tissue values as an additional tool for retrospective geolocation. It further emphasizes the importance of taking the spatial gradients of δ^15^N values into account when inferring trophic level from δ^15^N tissue values. Of course, it is also important to consider the diet composition and corresponding trophic level of predators and how this may vary within the population (e.g. among age-classes or between sexes) since there is an enrichment of 0.5–1 ‰ for δ^13^C values and 3–5 ‰ for δ^15^N values per trophic level [55, 76].

3. **Influence of foraging habitat on a species-specific δ**^**15**^**N isoscape**

Even though the δ^15^N plasma values of the sooty albatross did not show any correlation with latitude, the correlation between their δ^15^N plasma values and longitude may still provide evidence towards the second hypothesis (i.e. baseline δ^15^N gradient influence on marine predator δ^15^N tissue values). Foraging of sooty albatross breeding at Marion Island within 20–25° longitude is largely concentrated along the South West Indian Ridge [80]. The interaction between the fast-flowing Antarctic Circumpolar Current and this ridge results in zones of upwelling as well as meandering of fronts and eddy formation [81]. Consequently, pockets of high productivity all along the ridge may cause periodically higher δ^15^N values at the base of the food web [82]. This may explain the longitudinal gradient in the δ^15^N values between individual sooty albatross feeding closer to the ridge (between 20 and 25°E) and further downstream of it. Without concurrent stomach content samples of the tracked birds or samples of phytoplankton in the birds’ foraging areas, no definite conclusion can be drawn. Compound specific stable isotope analysis could be used in future investigations to overcome these uncertainties [83].

### Fine scale spatial variability in δ^13^C and δ^15^N isoscapes estimated from seabird tissues

This is the first study to combine the use of GPS dataloggers and the carbon and nitrogen stable isotope values of penguins to investigate fine-scale isotopic variability across space. It was found that even though all three penguin species stayed much closer to the island (0.5° latitudinal range) than the albatrosses and giant petrels (30° latitudinal range), they had lower δ^13^C plasma values than most of the flying seabirds (Table 1). This indicates that, while their isotopic values may be affected by latitudinal gradients in δ^13^C and δ^15^N stable isotopes (this study [14, 16, 20]), there are other factors impacting their isotopic values. These factors are most likely related to the major differences in foraging ecology between penguins and flying seabirds as well as regional physical features which contribute to fine scale spatial variability in baseline isotopic values at the Prince Edward Archipelago compared to elsewhere [18, 84, 85].

The inshore consumers at the Prince Edward Archipelago, like the gentoo penguin [86], derive their energy from external and internal sources. The external source is a result of allochthonous prey species being advected and concentrated around the archipelago by the Antarctic Circumpolar Current [87, 88]. Internally, due to the island mass effect, there is localized enhancement of biological productivity [87, 89]. Due to these two different energy sources, autochthonous species (e.g. microphytoplankton, benthic krill *Nauticaris marionis*, and fish), particularly benthic species, have higher values of both δ^13^C and δ^15^N compared to allochthonous species (e.g. sub-Antarctic krill *Euphausia vallentini*) found in the vicinity of the archipelago or in the surrounding pelagic waters [18, 84, 85].

The δ^13^C and δ^15^N isoscapes of penguins are therefore a combination of their diet and foraging habitat. The gentoo penguin breeding on Marion Island are strictly inshore foragers and are typically benthic foragers but they can also forage pelagically [86]. Their diet at Marion Island is highly variable, composed of mainly benthic fish and an autochthonous benthic crustacean (i.e. *Nauticaris marionis*) but can contain large proportions of pelagic crustaceans and juvenile fish if they are available [90, 91]. In contrast, the crested penguins (macaroni and eastern rockhopper penguins) are typically pelagic foragers with the eastern rockhopper penguin also exhibiting the ability to forage benthically [46, 53]. Although the brooding macaroni penguin reach further distances away from the island than the brooding eastern rockhopper penguin (this study [53]), the diet of both species is almost completely dominated by allochthonous pelagic krill species (i.e. *Euphausia vallentini* and *Thysanoessa vicina*), with small amounts of fish being eaten by both species ([92]; Pistorius unpub. data; Department of Environment, Forestry and Fisheries unpub. data). Thus, the inshore/offshore gradient in both δ^13^C and δ^15^N plasma values of penguins in this study is largely related to the benthic and autochthonous foraging ecology of the gentoo penguin and the pelagic, allochthonous foraging ecology of the crested penguins. Cherel and Hobson [17] similarly attributed the higher δ^13^C tissue values of shelf-feeding gentoo penguin at the Kerguelen Archipelago to local enhanced productivity and benthic feeding. In addition, although it has not been investigated for seabirds, there is the possibility of foraging depth having an impact on the isotopic values of penguins’ tissues, as seen in tunas [93].

## Conclusion

Using a multi-species approach this study shows that large and regional scale systematic spatial variability of δ^13^C and δ^15^N values at the base of the marine food web propagates through trophic levels and is reflected in the isotopic values of top predators’ tissues. This study provides evidence for the effect of baseline δ^15^N values on predator δ^15^N tissues values. This result emphasizes the importance of considering δ^15^N oceanic isoscapes in studies that incorporate stable isotopic values of marine predators to investigate their trophic ecology. Although these baseline values propagate through the food chain and are reflected across multiple marine predator species, they are not reflected in all species. This may be as a result of the movement of prey species or foraging of individuals across major water zones and fronts. It is therefore important when possible to estimate and apply species-specific isoscapes or have a good understanding of any factors and pathways affecting marine predators’ isotopic values when studying marine predator stable isotope ecology. It is further important to note that this study was performed using the stable isotope values of seabirds’ plasma, which may not reflect stable isotope values of other tissues such as whole blood or feathers [14]. With seasonal variation in the gradient of baseline δ^13^C highlighted in Magozzi et al. [16], future investigation should include intra- and inter-annual variability in stable isotope compositions detected at the top predator level.

## Supplementary information

**Additional file 1: Supplementary Material 1.** Approximate dates of different breeding stages throughout the year for wandering, grey-headed and sooty albatrosses, northern and southern giant petrels and gentoo, macaroni and eastern rockhopper penguins breeding at Marion Island, sub-Antarctic. Yellow dots represent approximate times when GPS data loggers were deployed on birds and blood plasma was collected for stable isotope analysis. **Supplementary Material 2.** Species-specific normalizing equations calculated from plasma samples (N) with both delipidated and raw plasma for wandering, grey-headed and sooty albatrosses and gentoo and macaroni penguins breeding at Marion Island, sub-Antarctic. **Supplementary Material 3.** Corresponding plasma a) δ^13^C (‰) and b) δ^15^N (‰) values given as mean ± SD (range) for different water zones and fronts (AZ: Antarctic zone, PF: polar front, PFZ: polar frontal zone, SAF: sub-Antarctic front, SAZ: sub-Antarctic zone, STF: sub-tropical front and STZ: sub-tropical zone) estimated from isoscapes interpolated from mean foraging locations and plasma isotopic values of wandering, grey-headed and sooty albatrosses, northern and southern giant petrels breeding at Marion Island.

## Data Availability

The datasets used and/or analysed during the current study are available from the corresponding author on reasonable request.

## Abbreviations

WA: Wandering albatross
GHA: Grey-headed albatross
SA: Sooty albatross
SGP: Southern giant petrel
NGP: Northern giant petrel
GP: Gentoo penguin
MP: Macaroni penguin
ERP: Eastern rockhopper penguin

## References

[1] Avila IC, Kaschner K, Dormann CF (2018). Current global risks to marine mammals: taking stock of the threats. Biol Conserv..

[2] Dias MP, Martin R, Pearmain EJ (2019). Threats to seabirds: a global assessment. Biol Conserv..

[3] Ropert-Coudert Y, Chiaradia A, Ainley D (2019). Happy feet in a hostile world? The future of penguins depends on proactive management of current and expected threats. Front Mar Sci.

[4] Ballard G, Jongsomjit D, Veloz SD, Ainley DG (2012). Coexistence of mesopredators in an intact polar ocean ecosystem: the basis for defining a Ross Sea marine protected area. Biol Conserv..

[5] Daly R, Smale MJ, Singh S (2018). Refuges and risks: evaluating the benefits of an expanded MPA network for mobile apex predators. Divers Distrib..

[6] Grémillet D, Boulinier T (2009). Spatial ecology and conservation of seabirds facing global climate change: a review. Mar Ecol Prog Ser..

[7] Harcourt R, Sequeira AMM, Zhang X (2019). Animal-borne telemetry: an integral component of the ocean observing toolkit. Front Mar Sci..

[8] Hays GC, Bailey H, Bograd SJ (2019). Translating marine animal tracking data into conservation policy and management. Trends Ecol Evol..

[9] Hobson KA, Barnett-Johnson R, Cerling TE, West J, Bowen G, Dawson T, Tu KP (2010). Using isoscapes to track animal migration. Isoscapes: understanding movement, pattern, and process on earth through isotope mapping.

[10] Ramos R, González-Solís J (2012). Trace me if you can: the use of intrinsic biogeochemical markers in marine top predators. Front Ecol Environ..

[11] Roscales JL, Gómez-Díaz E, Neves V, González-Solís J (2011). Trophic versus geographic structure in stable isotope signatures of pelagic seabirds breeding in the Northeast Atlantic. Mar Ecol Prog Ser..

[12] Graham BS, Koch PL, Newsome SD, West JB, Bowen GJ, Dawson TE, Tu KP (2010). Using isoscapes to trace the movements and foraging behavior of top predators in oceanic ecosystems. Isoscapes: understanding movement, pattern, and process on earth through isotope mapping.

[13] Connan M, McQuaid CD, Bonnevie BT (2014). Combined stomach content, lipid and stable isotope analyses reveal spatial and trophic partitioning among three sympatric albatrosses from the Southern Ocean. Mar Ecol Prog Ser..

[14] Jaeger A, Lecomte VJ, Weimerskirch H (2010). Seabird satellite tracking validates the use of latitudinal isoscapes to depict predators’ foraging areas in the Southern Ocean. Rapid Commun Mass Spectrom..

[15] Whitehead TO, Connan M, Ropert-Coudert Y, Ryan PG (2017). Subtle but significant segregation in the feeding ecology of sympatric penguins during the critical pre-moult period. Mar Ecol Prog Ser..

[16] Magozzi S, Yool A, Vander Zanden HB (2017). Using ocean models to predict spatial and temporal variation in marine carbon isotopes. Ecosphere..

[17] Cherel Y, Hobson KA (2007). Geographical variation in carbon stable isotope signatures of marine predators: a tool to investigate their foraging areas in the Southern Ocean. Mar Ecol Prog Ser..

[18] Kaehler S, Pakhomov EA, McQuaid CD (2000). Trophic structure of the marine food web at the Prince Edward islands (Southern Ocean) determined by δ^13^C and δ^15^N analysis. Mar Ecol Prog Ser..

[19] Quillfeldt P, McGill RAR, Furness RW (2005). Diet and foraging areas of Southern Ocean seabirds and their prey inferred from stable isotopes: review and case study of Wilson’s storm-petrel. Mar Ecol Prog Ser..

[20] Somes CJ, Schmittner A, Galbraith ED (2010). Simulating the global distribution of nitrogen isotopes in the ocean. Global Biogeochem Cy..

[21] Takai N, Onaka S, Ikeda Y (2000). Geographical variations in carbon and nitrogen stable isotope ratios in squid. J Mar Biol Assoc UK..

[22] Hobson KA, Clark RG (1993). Turnover of ^13^C in cellular and plasma fractions of blood: implications for non-destructive sampling in avian dietary studies. Auk..

[23] Brault EK, Koch PL, McMahon KW (2018). Carbon and nitrogen zooplankton isoscapes in West Antarctica reflect oceanographic transitions. Mar Ecol Prog Ser..

[24] Navarro J, Coll M, Somes CJ, Olson RJ (2013). Trophic niche of squids: insights from isotopic data in marine systems worldwide. Deep-Sea Res II..

[25] Francois R, Altabet MA, Goericke R (1993). Changes in the δ^13^C of surface water particulate organic matter across the subtropical convergence in the SW Indian Ocean. Global Biogeochem Cy..

[26] Lourey MJ, Trull TW, Sigman DM (2003). Sensitivity of δ^15^N of nitrate, surface suspended and deep sinking particulate nitrogen to seasonal nitrate depletion in the Southern Ocean. Global Biogeochem Cy..

[27] Trull TW, Armand L (2001). Insights into Southern Ocean carbon export from the δ^13^C of particles and dissolved inorganic carbon during the SOIREE iron release experiment. Deep-Sea Res II Top Stud Oceanogr..

[28] McMahon KW, Hamady LL, Thorrold SR (2013). Ocean ecogeochemistry: a review. Oceanogr Mar Biol An Annu Rev..

[29] Hoefs J (2015). Stable Isotope Geochemistry.

[30] O’Reilly CM, Verburg P, Hecky RE, Seuront L, Strutton P (2003). Food web dynamics in stable isotope ecology: time integration of different trophic levels. Handbook of scaling methods in aquatic ecology: measurement, analysis.

[31] Ceia FR, Cherel Y, Paiva VH, Ramos JA (2018). Stable isotope dynamics (δ^13^C and δ^15^N) in neritic and oceanic waters of the North Atlantic inferred from GPS-tracked Cory’s shearwaters. Front Mar Sci..

[32] Phillips RA, Croxall JP, Silk JRD, Briggs DR (2008). Foraging ecology of albatrosses and petrels from South Georgia: two decades of insights from tracking technologies. Aquat Conserv Mar Freshw Ecosyst..

[33] Cruz-Flores M, Militão T, Ramos R, Gonzàlez-Solis J (2018). Using marine isoscapes to infer movements of oceanic migrants: the case of Bulwer’s petrel, *Bulweria bulwerii*, in the Atlantic Ocean. PLoS One..

[34] Phillips RA, Bearhop S, McGill RAR, Dawson DA (2009). Stable isotopes reveal individual variation in migration strategies and habitat preferences in a suite of seabirds during the nonbreeding period. Oecologia..

[35] Bearhop S, Waldron S, Votier SC, Furness RW (2002). Factors that influence assimilation rates and fractionation of nitrogen and carbon stable isotopes in avian blood and feather. Physiol Biochem Zool..

[36] Harris S, Quintana F, Ciancio J, Riccialdelli L, Raya RA (2016). Linking foraging behavior and diet in a diving seabird. Mar Ecol..

[37] Hobson KA, Piatt JF, Pitocchelli J (1994). Using stable isotopes to determine seabird trophic relationships. J Anim Ecol..

[38] Ansorge IJ, Lutjeharms JRE (2002). The hydrography and dynamics of the ocean environment of the Prince Edward islands (Southern Ocean). J Mar Syst..

[39] Pakhomov EA, Froneman PW (1999). Macroplankton/ microneckton dynamics in the vicinity of the Prince Edward islands (Southern Ocean). Mar Biol..

[40] Ryan PG, Bester MN, Chown SL, Froneman PW (2008). Pelagic predators. The Prince Edward archipelago: land-sea interactions in a changing ecosystem.

[41] Hobson KA, Gibbs HL, Gloutney ML (1997). Preservation of blood and tissue samples for stable-carbon and stable-nitrogen isotope analysis. Can J Zool..

[42] Sumner MD (2016). trip: Tools for the analysis of animal track data. R package version 1.5.0.

[43] Baylis AMM, Tierney M, Orben RA (2019). Important at-sea areas of colonial breeding marine predators on the southern Patagonian shelf. Sci Rep..

[44] Calenge C (2006). The package “adehabitat” for the R software: a tool for the analysis of space and habitat use by animals. Ecol Model..

[45] Johnson DS, London JM, Lea MA, Durban JW (2008). Continuous-time correlated random walk model for animal telemetry data. Ecology..

[46] Tremblay Y, Cherel Y (2000). Benthic and pelagic dives: a new foraging behaviour in rockhopper penguins. Mar Ecol Prog Ser..

[47] Benhamou S (1992). Efficiency of area-concentrated searching behaviour in a continuous patchy environment. J Theor Biol..

[48] Pinaud D, Weimerskirch H (2007). At-sea distribution and scale-dependent foraging behaviour of petrels and albatrosses: a comparative study. J Anim Ecol..

[49] Garriga J, Palmer JRB, Oltra A, Bartumeus F (2016). Expectation–maximization binary clustering for behavioural annotation. PLoS One..

[50] de Grissac S, Bartumeus F, Cox SL, Weimerskirch H (2017). Early-life foraging: behavioral responses of newly fledged albatrosses to environmental conditions. Methods Ecol Evol..

[51] Jones TB, Patrick SC, Arnould JPY, Rodríguez-Malagón MA, Wells MR, Green JA (2018). Evidence of sociality in the timing and location of foraging in a colonial seabird. Biol Lett..

[52] Trathan PN, Bishop C, Maclean G, Brown P, Fleming A, Collins MA (2018). Linear tracks characterise penguin foraging pathways. Mar Ecol Prog Ser..

[53] Whitehead TO (2017). Comparative foraging ecology of macaroni and rockhopper penguins at the Prince Edward islands. University of Cape Town, South Africa. PhD thesis.

[54] Sato K, Charrassin JB, Bost CA, Naito Y (2004). Why do macaroni penguins choose shallow body angles that result in longer descent and ascent durations?. J Exp Biol..

[55] Post DM, Layman CA, Arrington DA (2007). Getting to the fat of the matter: models, methods and assumptions for dealing with lipids in stable isotope analyses. Oecologia..

[56] Cherel Y, Hobson KA, Weimerskirch H (2005). using stable isotopes to study resource acquisition and allocation in procellariiform seabirds. Oecologia..

[57] DeNiro MJ, Epstein S (1977). Mechanism of carbon isotope fractionation associated with lipid synthesis. Science..

[58] Logan JM, Jardine TD, Miller TJ (2008). Lipid corrections in carbon and nitrogen stable isotope analyses: comparison of chemical extraction and modelling methods. J Anim Ecol..

[59] Sotiropoulos MA, Tonn WM, Wassenaar LI (2004). Effects of lipid extraction on stable carbon and nitrogen isotope analyses of fish tissues: potential consequences for food web studies. Ecol Freshw Fish..

[60] Wackernagel H (1995). Ordinary kriging. Multivariate geostatics.

[61] St John Glew K, Graham LJ, Trueman CN, RAR MG (2019). Spatial models of carbon, nitrogen and sulphur stable isotope distributions (isoscapes) across a shrlf sea: An INLA approach. Methods Ecol Evol.

[62] Trueman CN, MacKenzie KM, St John GK (2017). Stable isotope-based location in a shelf sea setting: Accuracy and precision are comparable to light-based location methods. Methods Ecol Evol..

[63] Pebesma EJ (2004). Multivariable geostatistics in S: the gstat package. Comput Geosci..

[64] Hiemstra PH, Pebesma EJ, Twenhöfel CJW, Heuvelink GBM (2009). Real-time automatic interpolation of ambient gamma dose rates from the Dutch radioactivity monitoring network. Comput Geosci..

[65] Swart S, Speich S, Ansorge IJ, Lutjeharms JRE (2010). An altimetry-based gravest empirical mode south of Africa: 1. Development and validation. J Geophys Res.

[66] R Core Team (2020). R: A language and environment for statistical computing. R Foundation for Statistical Computing.

[67] Cherel Y, Klages NTW, Robertson G, Gales R (1998). A review of the food of albatrosses. Albatross biology and conservation.

[68] Nel DC, Lutjeharms JRE, Pakhomov EA (2001). Exploitation of mesoscale oceanographic features by grey-headed albatross *Thalassarche chrysostoma* in the southern Indian Ocean. Mar Ecol Prog Ser..

[69] Nel DC, Ryan PG, Nel JL (2002). Foraging interactions between wandering albatrosses (*Diomedea exulans*) breeding on Marion Island and long-line fisheries in the southern Indian Ocean. Ibis..

[70] Reisinger RR, Raymond B, Hindell MA (2018). Habitat modelling of tracking data from multiple marine predators identifies important areas in the Southern Indian Ocean. Divers Distrib..

[71] Hunter S, Brooke ML (1992). The diet of giant petrels *Macronectes* spp. at Marion Island, southern Indian Ocean. Colon Waterbirds..

[72] Cooper J, Klages NTW (1995). The diets and dietary segregation of sooty albatross (Phoebetria spp.) at subantarctic Marion Island. Antarct Sci.

[73] Naik RK, George JV, Soares MA (2015). Phytoplankton community structure at the juncture of the Agulhas Return Front and Subtropical Front in the Indian Ocean sector of Southern Ocean: Bottom-up and top-down control. Deep-Sea Res Pt II..

[74] Weimerskirch H (2007). Are seabirds foraging for unpredictable resources?. Deep-Sea Res II Top Stud Oceanogr..

[75] Bost C-A, Cotté C, Bailleul F (2009). The importance of oceanographic fronts to marine birds and mammals of the southern oceans. J Mar Syst..

[76] Michener RH, Schell DM, Michener LK (1994). Stable isotope ratios as tracers in marine aquatic food webs. Stable isotopes in ecology and environmental science.

[77] Carpenter-Kling T, Pistorius P, Connan M (2019). Sensitivity of δ^13^C values of seabird tissues to combined spatial, temporal and ecological drivers: a simulation approach. J Exp Mar Biol Ecol..

[78] Post DM (2002). Using stable isotopes to estimate trophic position: Models, methods, and assumptions. Ecology..

[79] Cherel Y, Hobson KA, Bailleul F, Groscolas A (2005). Nutrition, physiology, and stable isotopes: new information from fasting and molting penguins. Ecology..

[80] Schoombie S, Dilley BJ, Davies D (2017). The distribution of breeding sooty albatrosses from the three most important breeding sites : Gough, Tristan and the Prince Edward islands. Emu..

[81] Ansorge IJ, Lutjeharms JRE (2005). Direct observations of eddy turbulence at a ridge in the Southern Ocean. Geophys Res Lett..

[82] DiFiore PJ, Sigman DM, Trull TW (2006). Nitrogen isotope constraints on subantarctic biogeochemistry. J Geophys Res..

[83] Whiteman JP, Elliot Smith EA, Besser AC, Newsome SD (2019). A guide to using compund-specific stable isotope analysis to study the fates of molecules in organisms and ecosystems. Diversity..

[84] Allan EL, Froneman PW, Durgadoo JV (2013). Critical indirect effects of climate change on sub-Antarctic ecosystem functioning. Ecol Evol..

[85] Pakhomov EA, McClelland JW, Bernard K (2004). Spatial and temporal shifts in stable isotope values of the bottom-dwelling shrimp *Nauticaris marionis* at the sub-Antarctic archipelago. Mar Biol..

[86] Carpenter-Kling T, Handley JM, Green DB (2017). A novel foraging strategy in gentoo penguins breeding at sub-Antarctic Marion Island. Mar Biol..

[87] Ansorge IJ, Froneman PW, Pakhomov EA (1999). Physical-biological coupling in the waters surrounding the Prince Edward islands (Southern Ocean). Polar Biol..

[88] Perissinotto R, McQuaid CD (1992). Land-based predator impact on vertically migrating zooplankton and micronekton advected to a Southern Ocean archipelago. Mar Ecol Prog Ser..

[89] Perissinotto R, Duncombe Rae CM (1990). Occurance of anticyclonic eddies on the Prince Edward plateau (Southern Ocean): effects on phytoplankton biomass and production. Deep Sea Res A..

[90] Adams NJ, Klages NTW (1989). Temporal variation in the diet of the gentoo penguin *Pygoscelis papua* at sub-Antarctic Marion Island. Colon Waterbirds..

[91] Carpenter-Kling T, Handley JM, Connan M (2019). Gentoo penguins as sentinels of climate change at the sub-Antarctic Prince Edward archipelago, Southern Ocean. Ecol Indic.

[92] Brown CR, Klages NTW (1987). Seasonal and annual variation in the diets of macaroni (*Eudyptes chrysolophus*) and southern rockhopper (*E. chyrsocome chrysocome*) penguins a sub-antarctic Marion Island. J Zool Lond..

[93] Houssard P, Lorrain A, Tremblay-Boyer L (2017). Trophic position increases with thermocline depth in yellowfin and bigeye tuna across the Western and Central Pacific Ocean. Prog Oceanogr..

