# Supplementary material for: A critical assessment of marine predator isoscapes within the southern Indian Ocean
Source: Mov Ecol. 2020 Jun 29;8:29. doi: 10.1186/s40462-020-00208-8 (PMC7322845; doi:10.1186/s40462-020-00208-8)
Supplement: Supplementary file 1 — Additional file 1: Supplementary Material 1. Approximate dates of different breeding stages throughout the year for wandering, grey-headed and sooty albatrosses, northern and southern giant petrels and gentoo, macaroni and eastern rockhopper penguins breeding at Marion Island, sub-Antarctic. Yellow dots represent approximate times when GPS data loggers were deployed on birds and blood plasma was collected for stable isotope analysis. Supplementary Material 2. Species-specific normalizing equations calculated from plasma samples (N) with both delipidated and raw plasma for wandering, grey-headed and sooty albatrosses and gentoo and macaroni penguins breeding at Marion Island, sub-Antarctic. Supplementary Material 3. Corresponding plasma a) δ13C (‰) and b) δ15N (‰) values given as mean ± SD (range) for different water zones and fronts (AZ: Antarctic zone, PF: polar front, PFZ: polar frontal zone, SAF: sub-Antarctic front, SAZ: sub-Antarctic zone, STF: sub-tropical front and STZ: sub-tropical zone) estimated from isoscapes interpolated from mean foraging locations and plasma isotopic values of wandering, grey-headed and sooty albatrosses, northern and southern giant petrels breeding at Marion Island. [file 40462_2020_208_MOESM1_ESM.pptx]

## Slide 1
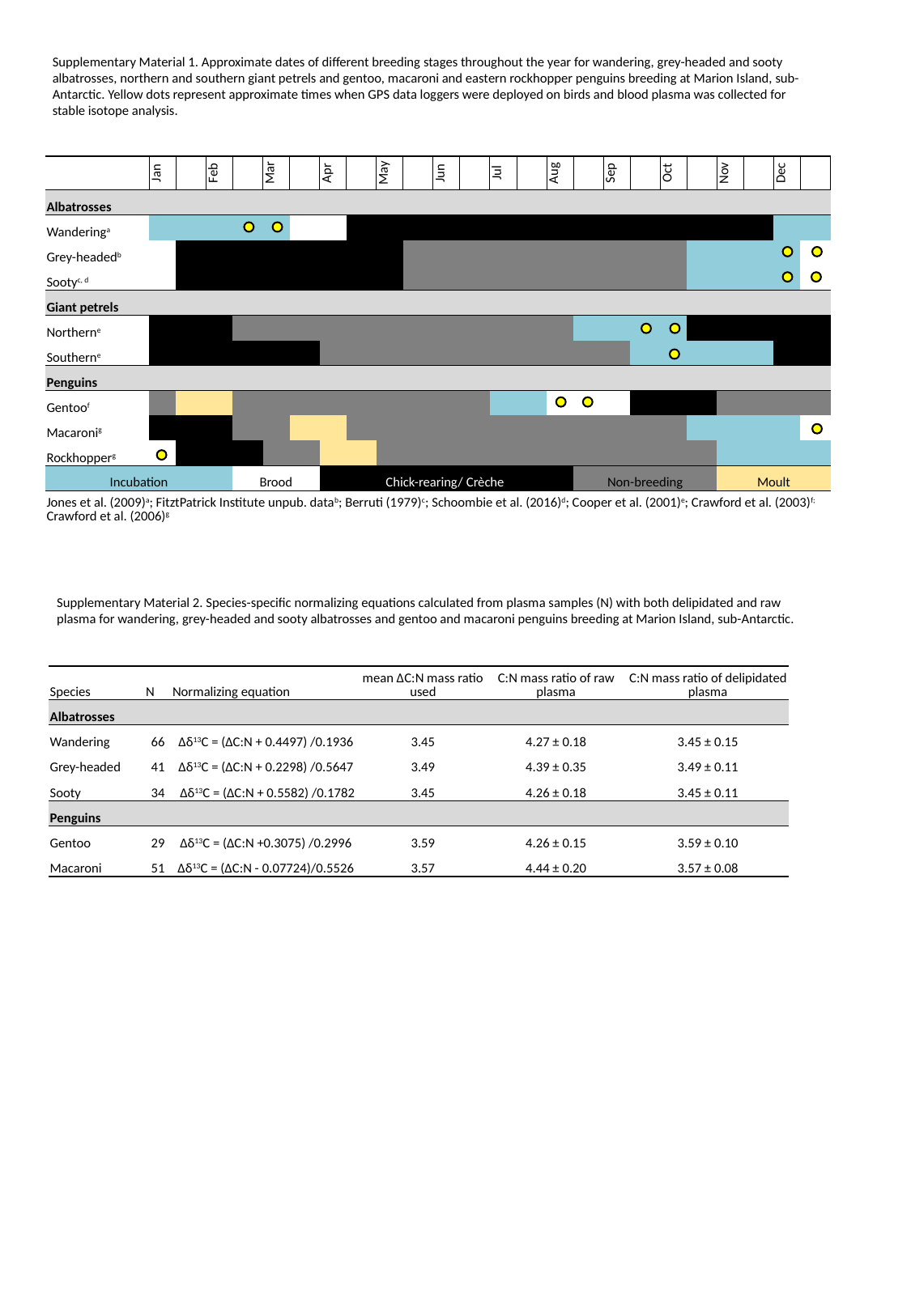

Supplementary Material 1. Approximate dates of different breeding stages throughout the year for wandering, grey-headed and sooty albatrosses, northern and southern giant petrels and gentoo, macaroni and eastern rockhopper penguins breeding at Marion Island, sub-Antarctic. Yellow dots represent approximate times when GPS data loggers were deployed on birds and blood plasma was collected for stable isotope analysis.
| | Jan | | Feb | | Mar | | Apr | | May | | Jun | | Jul | | Aug | | Sep | | Oct | | Nov | | Dec | |
| --- | --- | --- | --- | --- | --- | --- | --- | --- | --- | --- | --- | --- | --- | --- | --- | --- | --- | --- | --- | --- | --- | --- | --- | --- |
| Albatrosses | | | | | | | | | | | | | | | | | | | | | | | | |
| Wanderinga | | | | | | | | | | | | | | | | | | | | | | | | |
| Grey-headedb | | | | | | | | | | | | | | | | | | | | | | | | |
| Sootyc, d | | | | | | | | | | | | | | | | | | | | | | | | |
| Giant petrels | | | | | | | | | | | | | | | | | | | | | | | | |
| Northerne | | | | | | | | | | | | | | | | | | | | | | | | |
| Southerne | | | | | | | | | | | | | | | | | | | | | | | | |
| Penguins | | | | | | | | | | | | | | | | | | | | | | | | |
| Gentoof | | | | | | | | | | | | | | | | | | | | | | | | |
| Macaronig | | | | | | | | | | | | | | | | | | | | | | | | |
| Rockhopperg | | | | | | | | | | | | | | | | | | | | | | | | |
| Incubation | | | | Brood | | | Chick-rearing/ Crèche | | | | | | | | | Non-breeding | | | | | Moult | | | |
| Jones et al. (2009)a; FitztPatrick Institute unpub. datab; Berruti (1979)c; Schoombie et al. (2016)d; Cooper et al. (2001)e; Crawford et al. (2003)f; Crawford et al. (2006)g | | | | | | | | | | | | | | | | | | | | | | | | |
Supplementary Material 2. Species-specific normalizing equations calculated from plasma samples (N) with both delipidated and raw plasma for wandering, grey-headed and sooty albatrosses and gentoo and macaroni penguins breeding at Marion Island, sub-Antarctic.
| Species | N | Normalizing equation | mean ∆C:N mass ratio used | C:N mass ratio of raw plasma | C:N mass ratio of delipidated plasma |
| --- | --- | --- | --- | --- | --- |
| Albatrosses | | | | | |
| Wandering | 66 | ∆δ13C = (∆C:N + 0.4497) /0.1936 | 3.45 | 4.27 ± 0.18 | 3.45 ± 0.15 |
| Grey-headed | 41 | ∆δ13C = (∆C:N + 0.2298) /0.5647 | 3.49 | 4.39 ± 0.35 | 3.49 ± 0.11 |
| Sooty | 34 | ∆δ13C = (∆C:N + 0.5582) /0.1782 | 3.45 | 4.26 ± 0.18 | 3.45 ± 0.11 |
| Penguins | | | | | |
| Gentoo | 29 | ∆δ13C = (∆C:N +0.3075) /0.2996 | 3.59 | 4.26 ± 0.15 | 3.59 ± 0.10 |
| Macaroni | 51 | ∆δ13C = (∆C:N - 0.07724)/0.5526 | 3.57 | 4.44 ± 0.20 | 3.57 ± 0.08 |

## Slide 2
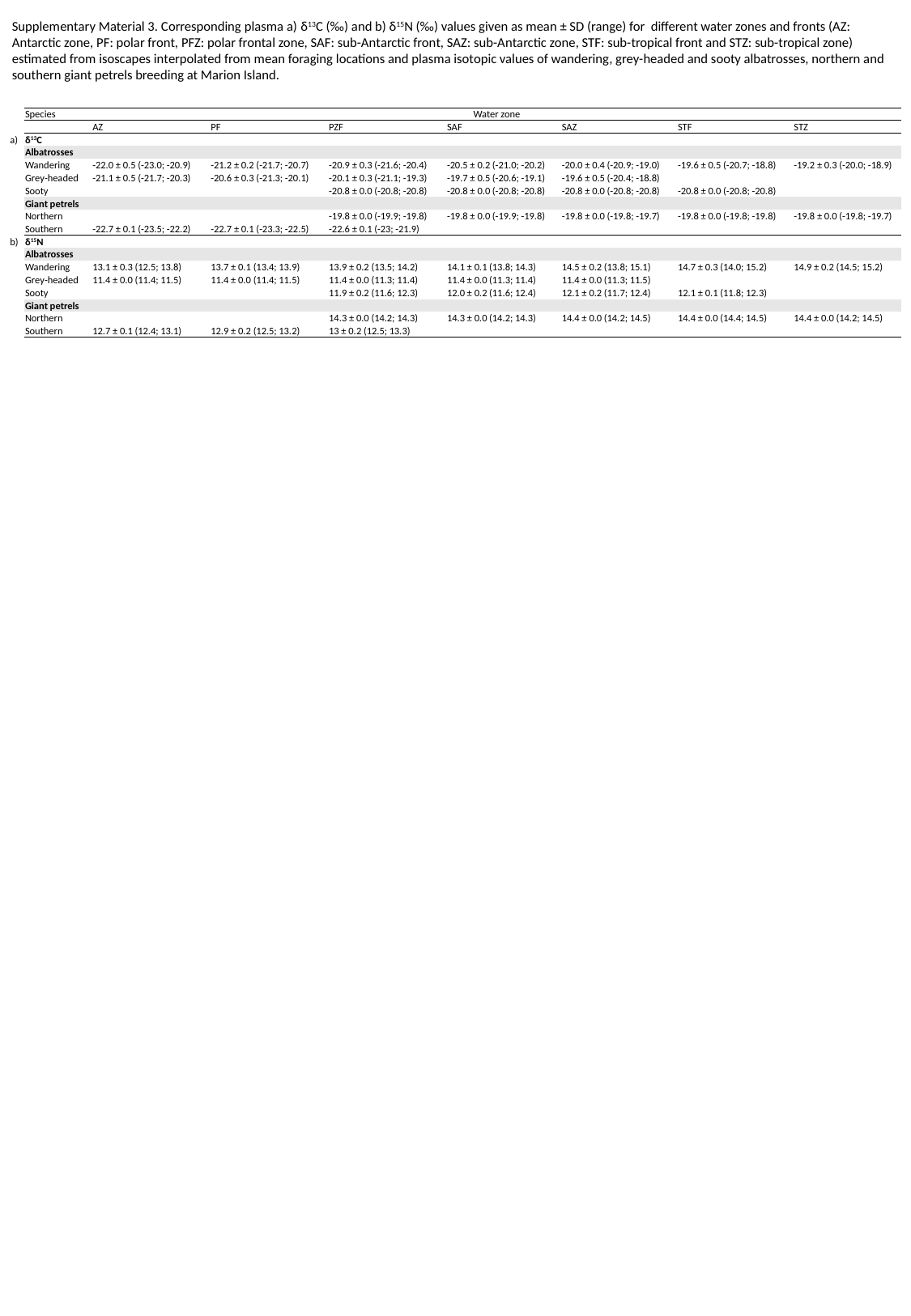

Supplementary Material 3. Corresponding plasma a) δ13C (‰) and b) δ15N (‰) values given as mean ± SD (range) for different water zones and fronts (AZ: Antarctic zone, PF: polar front, PFZ: polar frontal zone, SAF: sub-Antarctic front, SAZ: sub-Antarctic zone, STF: sub-tropical front and STZ: sub-tropical zone) estimated from isoscapes interpolated from mean foraging locations and plasma isotopic values of wandering, grey-headed and sooty albatrosses, northern and southern giant petrels breeding at Marion Island.
| | Species | Water zone | | | | | | |
| --- | --- | --- | --- | --- | --- | --- | --- | --- |
| | | AZ | PF | PZF | SAF | SAZ | STF | STZ |
| a) | δ13C | | | | | | | |
| | Albatrosses | | | | | | | |
| | Wandering | -22.0 ± 0.5 (-23.0; -20.9) | -21.2 ± 0.2 (-21.7; -20.7) | -20.9 ± 0.3 (-21.6; -20.4) | -20.5 ± 0.2 (-21.0; -20.2) | -20.0 ± 0.4 (-20.9; -19.0) | -19.6 ± 0.5 (-20.7; -18.8) | -19.2 ± 0.3 (-20.0; -18.9) |
| | Grey-headed | -21.1 ± 0.5 (-21.7; -20.3) | -20.6 ± 0.3 (-21.3; -20.1) | -20.1 ± 0.3 (-21.1; -19.3) | -19.7 ± 0.5 (-20.6; -19.1) | -19.6 ± 0.5 (-20.4; -18.8) | | |
| | Sooty | | | -20.8 ± 0.0 (-20.8; -20.8) | -20.8 ± 0.0 (-20.8; -20.8) | -20.8 ± 0.0 (-20.8; -20.8) | -20.8 ± 0.0 (-20.8; -20.8) | |
| | Giant petrels | | | | | | | |
| | Northern | | | -19.8 ± 0.0 (-19.9; -19.8) | -19.8 ± 0.0 (-19.9; -19.8) | -19.8 ± 0.0 (-19.8; -19.7) | -19.8 ± 0.0 (-19.8; -19.8) | -19.8 ± 0.0 (-19.8; -19.7) |
| | Southern | -22.7 ± 0.1 (-23.5; -22.2) | -22.7 ± 0.1 (-23.3; -22.5) | -22.6 ± 0.1 (-23; -21.9) | | | | |
| b) | δ15N | | | | | | | |
| | Albatrosses | | | | | | | |
| | Wandering | 13.1 ± 0.3 (12.5; 13.8) | 13.7 ± 0.1 (13.4; 13.9) | 13.9 ± 0.2 (13.5; 14.2) | 14.1 ± 0.1 (13.8; 14.3) | 14.5 ± 0.2 (13.8; 15.1) | 14.7 ± 0.3 (14.0; 15.2) | 14.9 ± 0.2 (14.5; 15.2) |
| | Grey-headed | 11.4 ± 0.0 (11.4; 11.5) | 11.4 ± 0.0 (11.4; 11.5) | 11.4 ± 0.0 (11.3; 11.4) | 11.4 ± 0.0 (11.3; 11.4) | 11.4 ± 0.0 (11.3; 11.5) | | |
| | Sooty | | | 11.9 ± 0.2 (11.6; 12.3) | 12.0 ± 0.2 (11.6; 12.4) | 12.1 ± 0.2 (11.7; 12.4) | 12.1 ± 0.1 (11.8; 12.3) | |
| | Giant petrels | | | | | | | |
| | Northern | | | 14.3 ± 0.0 (14.2; 14.3) | 14.3 ± 0.0 (14.2; 14.3) | 14.4 ± 0.0 (14.2; 14.5) | 14.4 ± 0.0 (14.4; 14.5) | 14.4 ± 0.0 (14.2; 14.5) |
| | Southern | 12.7 ± 0.1 (12.4; 13.1) | 12.9 ± 0.2 (12.5; 13.2) | 13 ± 0.2 (12.5; 13.3) | | | | |
